# The Volatile Organic Compounds of *Streptomyces* spp.: An In-Depth Analysis of Their Antifungal Properties

**DOI:** 10.3390/microorganisms11071820

**Published:** 2023-07-16

**Authors:** Lorena Cuervo, Samuel Álvarez-García, José A. Salas, Carmen Méndez, Carlos Olano, Mónica G. Malmierca

**Affiliations:** 1 Functional Biology Department, University of Oviedo, 33006 Oviedo, Spain; cuervolorena@uniovi.es (L.C.); jasalas@uniovi.es (J.A.S.); cmendezf@uniovi.es (C.M.); olanocarlos@uniovi.es (C.O.); 2 University Institute of Oncology of Asturias (I.U.O.P.A), University of Oviedo, 33006 Oviedo, Spain; 3 Health Research Institute of Asturias (ISPA), Av. del Hospital Universitario, s/n, 33011 Oviedo, Spain; 4 Plant Physiology Area, Engineering and Agricultural Sciences Department, Universidad de León, 24009 León, Spain; salvg@unileon.es

**Keywords:** *Streptomyces*, antifungal, GS-MS, *Escovopsis weberi*, volatile organic compounds, secondary metabolism

## Abstract

The study of volatile organic compounds (VOCs) has expanded because of the growing need to search for new bioactive compounds that could be used as therapeutic alternatives. These small molecules serve as signals to establish interactions with other nearby organisms in the environment. In this work, we evaluated the antifungal effect of VOCs produced by different *Streptomyces* spp. This study was performed using VOC chamber devices that allow for the free exchange of VOCs without physical contact between microorganisms or the diffusible compounds they produce. Antifungal activity was tested against *Escovopsis weberi,* a fungal pathogen that affects ant nest stability, and the results showed that *Streptomyces* spp. CS014, CS057, CS131, CS147, CS159, CS207, and CS227 inhibit or reduce the fungal growth with their emitted VOCs. A GS-MS analysis of volatiles produced and captured by activated charcoal suggested that these *Streptomyces* strains synthesize several antifungal VOCs, many of them produced because of the presence of *E. weberi,* with the accumulation of various VOCs determining the growth inhibition effect.

## 1. Introduction

Actinomycetes are a group of Gram-positive bacteria commonly found in soils. Soil is a complex habitat exposed to highly variable conditions and colonized by a great number of species that interact and compete with each other. In this scenario, intra- and interspecific interactions play a key role in the stability and evolution of the biological community living below ground. The most studied interactions are based on the secretion of water-soluble compounds that can act as attractants, serve as alarm signals against predators, confer a competitive advantage, or even kill the recipient organisms [1] Among the Actinomycetes, bacteria of the genus *Streptomyces* stand out from a biotechnological point of view because of their enormous biosynthetic potential [2,3]; in fact, they are responsible for the production of many of the bioactive compounds used today in human and animal health [4]. *Streptomyces* has evolved over time to adapt and survive in such a competitive environment by producing a number of specialized metabolites. To overcome abiotic stresses, the vast majority of *Streptomyces* species produce siderophores, which provide them with a nutritional advantage over other organisms by solubilizing soil metals, thus increasing their availability, photoprotective compounds (such as melanin or carotenes), and osmoprotective metabolites (such as ectoin) [5]. Another competitive advantage of these bacteria is their significant ability to produce a wide array of diffusible compounds with antibacterial, antifungal, and antiviral activity (e.g., cosmomycin, nystatin, and valinomycin, respectively). These metabolites, with different biosynthetic origins, allow them to compete for space and nutrients and colonize new environments by displacing previously established species [6].

Since most soils are not saturated with water, the dispersion of water-soluble compounds is restricted to short distances. Indeed, the large number of air-filled soil pores suggests the important role volatile organic compounds (VOCs) can play in this habitat, covering much greater distances than soluble compounds [7]. Volatiles comprise a wide range of metabolites belonging to different chemical classes but with common features: low molecular mass, high vapor pressure, a low boiling point, and a lipophilic nature [8]. In recent years, the study of microbial volatile organic compounds (MVOCs) has drawn the attention of the research community to their potential use in the biocontrol of agricultural pests, and the number of identified VOCs is rapidly increasing [9]. *Streptomyces* spp. are well-known producers of VOCs. Systematic surveys were conducted that led to the identification of more than 1400 VOCs produced by *Streptomyces* spp. belonging to different chemical classes and exerting diverse activities [6,10,11]. MVOCs are involved in important phenomena like quorum sensing/quenching, antibiosis, and/or communication; they can be assimilated into organic matter or even influence physiological processes (e.g., nitrification) [12,13].

The study of microbial volatiles is a promising new field that has suffered because of a lack of standardized, affordable, and simple equipment and methodologies because of the intrinsic nature of these compounds. Two main approaches are used to study MVOCs: passive diffusion systems, where volatiles diffuse freely into the headspace, and dynamic air stream systems, where compounds are funneled from the producer to the receiving organism, each with its own advantages and disadvantages [14]. Within the passive systems applied to bacterial VOCs, the divided Petri dish, plate-within-plate, and “sandwiched” Petri plate methods are the most used. Although they have been successfully applied in many studies, these methods have some drawbacks because of the high risk of cross-contamination and a lack of homogeneity and reproducibility in the results, and they can be difficult to set up. In this regard, new devices called volatile organic compound chambers (VOC chambers) were recently developed to solve these problems when working with microbial VOCs and demonstrated their usefulness and reliability in studying the antifungal VOCs produced by the filamentous fungus *Trichoderma* sp. [15]. This system was also successfully tested using *Streptomyces* spp. in our laboratory [16].

The urgent need for new bioactive compounds has led to the search for new producers in underexplored environments or symbiosis with other organisms [17,18]. Leafcutter ants are known to establish a complex relationship with various bacteria that help them fight the pathogenic fungus *Escovopsis weberi*. Bacteria of the genus *Pseudonocardia* have been described as true symbionts of these ants. However, a wide variety of *Streptomyces* strains have been found to co-inhabit ant nests. Much work has been performed describing the ability of these *Streptomyces* spp. to produce bioactive compounds (e.g., candicidins, actinomycins, sipanmycins) that could be added to those produced by *Pseudonocardia* to defend nests against other microorganisms [18,19]. The nests of these ants have a complex architecture made up of different underground chambers. This closed environment seems to be an ideal scenario for volatile compounds to have great relevance.

We hypothesize that VOCs emitted by *Streptomyces* sp. strains isolated from leafcutters impair the development of the pathogenic fungus *E. weberi.* We used the VOC chamber developed by Álvarez-García and co-workers to study VOCs produced by physically separated species and applied it, for the first time, to the analysis of interactions between *Streptomyces* spp. And *E. weberi*, mediated by volatile compounds. We found that several of these bacterial strains are capable of inhibiting (or delaying) the growth of the fungus in vitro. Also, we analyzed the VOCs present during that interaction using GC-MS and identified the possible volatile compounds responsible for the observed antifungal activity.

## 2. Materials and Methods

### 2.1. Strains and Culture Conditions

*Streptomyces* sp. strains used in this work belong to an in-lab CS collection (named after Carlos Sialer) isolated from the cuticle of leafcutter ants from the tribe *Attini* [19,20]. Strains were routinely grown on MA plates (Medium A) [21]) and incubated at 28 °C for 7 days. For metabolite production, strains were grown on either R5A [22] or soy flour mannitol medium (SFM) [23] agar plates. The pathogenic fungi *E. weberi* strain E and *Sclerotinia* spp. were cultivated at 28 °C on PDA plates (Potato Dextrose Agar, Oxoid), allowing for confluent growth, and then spread onto a fresh PDA plate with a cotton wool bud. For *Phytophthora cinnamomi* culturing, Sabouraud (Scharlab) medium was used. For multiple cocultures, SFM and YMA media (yeast extract, 3 g; malt extract, 3 g; peptone, 5 g, and glucose, 10 g per liter) were used.

### 2.2. Cocultured Streptomyces spp.–E. weberi

Spores of *Streptomyces* spp. were spread on half an R5A, SFM, or PDA plate. At the extreme of the other half of the plate, an agar plug of grown *E. weberi* was set as a fungi inoculum. After 5 days of incubating at 28 °C, we observed whether the fungus exceeded the confrontation line. Each experiment was performed in triplicate. The same experiment was repeated using *E. weberi* agar plugs confronted with two-day-grown *Streptomyces* spp.

### 2.3. Dual-Cultured Streptomyces spp.–E. weberi in VOC Chambers

**Figure 1:**
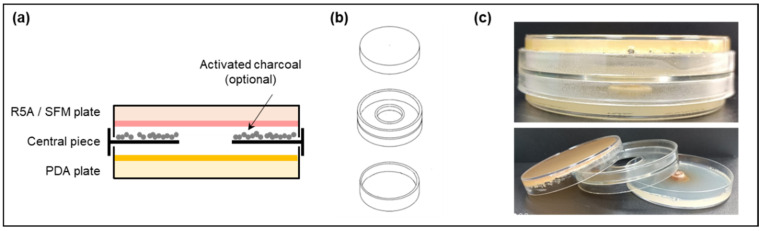
Volatile organic compound (VOC) chamber. (**a**) Schematic side view of a VOC chamber; (**b**) representation of each part of the VOC chamber device. The hole in the middle allows for the exchange of VOCs between cultures (modified from [16]); (**c**) photographs of an assembled VOC chamber (without charcoal).

*Streptomyces* sp. strains were grown on R5A or SFM plates at 28 °C for two days. An agar plug from the edge of a growing colony of *E. weberi* was placed in the center of a PDA plate, and immediately, a VOC chamber device was mounted as follows: a vented central piece was placed on top of the PDA plate (with the fungal agar plug facing up), and the plate with the two-day-grown *Streptomyces* spp. was placed upside down on top of it (Figure 1). The chamber device had a hole in the central part (without any type of film or filter covering it), allowing for the free exchange of VOCs between the cultures and avoiding physical contact between the strains or with the compounds that diffuse into the medium. The assembled VOC chamber was sealed with Parafilm^®^ (Bemis, E-Thermo Fisher Scientific, Madrid, Spain). The dual culture was incubated at 28 °C for 5 days, and the diameter of the fungal colony was recorded after 2 and 5 days.

The *E. weberi* colony diameter (in mm and measured in two different directions) was considered the mean value of the aforementioned two measurements. The percentage of fungal growth inhibition (GI) was calculated following the formula %GI = [(C − T)/C] × 100, where C is the colony size cultivated in the control plate, and T is the colony size measured in the VOC chamber. Each experiment was conducted in triplicate (*n* = 3). Control VOC chambers were also set without the *E. weberi* or the *Streptomyces* sp. strains.

### 2.4. Capture of VOCs and GC-MS Analysis

To analyze the compounds produced by the dual culture in SFM or R5A media, 250 mg of activated charcoal (Norit^®^) was placed on top of the chamber´s central piece, aiming to capture the emitted VOCs. This methodology was also applied to analyze VOCs produced by a single culture of *E. weberi* and a single culture of *Streptomyces* spp. in SFM or R5A media (only for strains CS057, CS131, CS014, and CS147). A VOC environmental control, an empty plate with active carbon, was also arranged. After 5 days of incubation at 28 °C, the activated charcoal was removed, and the captured volatiles were extracted with 750 µL of ethyl acetate. Also, an ethyl acetate sample was analyzed as a control to evaluate the volatile compounds that could be present in the solvent. Afterward, these samples were analyzed via gas chromatography–mass spectrometry (GC-MS) using an Agilent Technologies 7890A GC System coupled with a 5975C Inert XL MSD mass spectrometer in SCAN mode. Gas chromatography was carried out on an Agilent DB-5MS column (30 m × 0.25 mm × 0.25 µm with helium as carrier gas at 1 mL/min). The initial oven temperature was 50 °C, held for 5 min, ramped at 5 °C/min up to 300 °C, and held for 20 min. MassHunter Unknowns Analysis software and the NIST20 mass spectral library were used to identify compounds with a high percentage of reliability.

### 2.5. Data Analysis

Compounds identified with more than 70% reliability were selected. In order to create graphics, Rstudio and UpsetR package were used.

### 2.6. Dual Cultures against Other Fungi

An agar plug from the edge of a growing colony of *Phytophthora cinnamomic* or *Sclerotinia* spp. was placed in the center of a Sabouraud agar and PDA plates, respectively. The VOC chamber device was mounted as previously described. Two-day-grown *Streptomyces* spp. were placed upside down on top of the chamber.

### 2.7. Analysis of Diffusible Compound Production

In total, 3.5 g of *Streptomyces* sp. agar plates grown on SFM medium cocultures with *E. weberi* or *P. cinnamomi* were extracted with 5 mL of different organic solvents (ethyl acetate, ethyl acetate containing formic acid (1%), or butanol) and analyzed via reverse phase chromatography in a Waters Acquity UPLC instrument fitted with a BEHC18 column (1.7 μm, 2.1 mm × 100 mm; Waters, Milford, MA, USA), with acetonitrile and MQ water + 0.1% trifluoroacetic acid (TFA) as the mobile phase. The photodiode array (PDA) detector was set to scan wavelengths between 200 and 600 nm. The samples were eluted with acetonitrile (10%) for 1 min, followed by a linear gradient of acetonitrile (10–100%) for 7 min (flow rate of 0.5 mL/min; column temperature, 35 °C). As a control for the assay, *Streptomyces* sp. agar plates without confrontations from any microorganisms were extracted.

### 2.8. Multiple-Cultured Streptomyces spp.–E.weberi

Two different *Streptomyces* spp. were cultured on small YMA or SFM Petri dishes (5 cm diameter). After 48 h, both plates and a small PDA plate containing an agar plug from the edge of a growing colony of *E. weberi* were placed open inside a large Petri dish (diameter of 13.5 cm). This device was sealed with Parafilm^®^ (Bemis, E-Thermo Fisher Scientific, Madrid, Spain) and incubated at 28 °C for 5 days (Figure S1, Supplementary Material). The diameter of the fungal colony was recorded after 2 and 5 days. As a control, one *Streptomyces* sp. plate and an *E. weberi* plate were settled on a large one for confrontation. Moreover, an *E. weberi* dish alone was cultured on a large plate. Each experiment was conducted in triplicate.

### 2.9. pH Variation Analysis

To test the variation in the pH of the device environment as a consequence of the effect caused by VOCs, three different small and opened plates were placed inside a large one: an SFM *Streptomyces* sp. plate, a PDA plate with a plug of *E. weberi,* and a PDA + phenol red broth (Condalab) plate as a pH indicator. This device was incubated at 28 °C for 5 days. As a control, a *Streptomyces* sp. plate was cultured at 28 °C for 5 days in the presence of one plate containing the indicator. In addition, a single indicator plate and an *E. weberi* plate were placed inside the large dishes and incubated at 28 °C for 5 days.

## 3. Results and Discussion

The broad relationship described between the *Attini* ant tribe and *Streptomyces* spp. has left evidence of the important role that *Streptomyces* spp. might play in their nests. Their great potential to produce bioactive compounds suggests a benefit that the entire community can obtain for the maintenance of the system´s balance. One of the elements of the imbalance that threatens this system is the pathogen *E. weberi,* so it is worth investigating the involvement of *Streptomyces* in the biosynthesis of antifungal compounds that hold *E. weberi* at bay.

### 3.1. Cocultured Streptomyces spp.–E. weberi

This assay was performed in order to evaluate the effect of the coculture between *E. weberi* and different *Streptomyces* sp. strains of the CS collection. It was performed by confronting the fungus and the bacteria at the same time and confronting *E. weberi* with a two-day-grown *Streptomyces* sp. to evaluate if there are differences between the two setups.

The results of this experiment were variable. Three media were used: R5A and SFM, rich media for the growth of *Streptomyces* spp., and PDA, a suitable medium for *E. weberi* growth. However, a diversity of results was perceived depending on the strain, media, and whether the *E. weberi* plug was inoculated at the same time as the *Streptomyces* spp. or in a two-day-grown plate. This difference is based on the stationary phase of the two-day-grown *Streptomyces* spp., which allows these bacteria to produce secondary metabolites with possible antifungal properties [24]. However, in cases where both microorganisms are inoculated at the same time, this advantage of the *Streptomyces* sp. strain does not exist, and therefore, *E. weberi*, which grows faster, tends to overgrow and spread throughout the entire plate in most cases, even on top of the *Streptomyces* spp. culture (Figure S2, Supplementary Material).

In the case of cultures performed on PDA medium (which is more favorable for fungal growth), *E. weberi* grows throughout the entire plate, lysing most of the different *Streptomyces* sp. colonies tested, regardless of whether *Streptomyces* spp. were growing for 2 days before or not. In the case of two-day-grown CS159, this strain prevents *E. weberi* growth beyond the confronting line (the line delimiting the *Streptomyces* sp. growth zone). This fact does not occur when both microorganisms are grown at the same time, since *E. weberi* spreads over the entire surface of the plate. In the case of CS081a, CS147, and CS149 two-day-grown plates, a partial contention on the *E. weberi* growth can also be observed (Figure S2, Supplementary Material).

When the coculture occurs in R5A medium, it is favorable for *Streptomyces* spp. growth and the production of secondary metabolites because of the richness of its composition; the inhibition of *E. weberi* growth can be observed to a greater extent since it does not reach the confrontation line in any case (both in the plates with two-day-grown *Streptomyces* spp. and the ones with both microorganisms cultured at the same time). In some cases, it can be seen that this inhibition of growth is greater in the two-day-grown plates, for example, with strains CS090a and CS113 (Figure S2, Supplementary Material). This effect may be mainly due to the effect of diffusible compounds, as well as the volatiles produced by the *Streptomyces* sp. strains since they all have a great metabolic potential for the synthesis of antimicrobial compounds [5,25].

SFM is the medium that offers the most variable results. On the one hand, in cocultures with strains CS090a, CS113, CS207, and CS227, *E. weberi* exceeds the confrontation line, contrary to the results on R5A medium, where the growth of *E. weberi* is controlled by *Streptomyces* spp. This may be because the fungal growth on R5A may be more retarded or because antifungal metabolites that exert an inhibitory effect are biosynthesized in R5A and are not produced on SFM. However, in the majority of cases, the growth of *E. weberi* does not reach the confrontation line, but this inhibition is more pronounced in the case of the two-day-grown *Streptomyces* spp. (except for strains CS057, CS81a, and CS149). This seems to be logical; since the production of potential antifungal compounds by *Streptomyces* spp. occurs before the incorporation of *E. weberi* and therefore the inhibition is observed to a greater extent. In the case of the aforementioned exceptions, this effect is probably due to the production of variable compounds in the different stages of *Streptomyces* development (Figure S2, Supplementary Material) [26].

Based on these results, interest in the implications of volatile compounds in this assay increases. Indeed, in the natural environment in which these microorganisms coexist, both liquid and volatile compounds can be responsible for exerting an effect on the pathogen. However, it might also be true that the two types of microorganisms are not necessarily always in physical contact with each other, or the diffusible compounds do not have enough range of activity to affect *E. weberi,* so the effect of volatile compounds may play a more important role that we initially imagined. For that reason, a study of the effect of the volatile compounds of *Streptomyces* spp. on *E. weberi* was planned, with no contact between either microorganism or with the soluble compounds that they disseminate.

### 3.2. Dual-Cultured Streptomyces spp.–E. weberi in VOC Chambers

Coculture trials of the pathogenic fungus *E. weberi* and various strains of *Streptomyces* spp. from the CS collection were performed with the aim of evaluating the potential of the ecological relationships that these species, coexisting in the same environment, can maintain.

The use of VOC chambers is useful in evaluating signaling and effects between these two microorganisms because of the exclusive effect of the VOCs since there is no physical contact between them or with the compounds that both can diffuse into culture media. This strategy is important in determining that, although both species live in the same environment, there may not be physical contact between them, but the signaling relationships between them are maintained through volatiles, which can trigger different types of effects.

**Figure 2:**
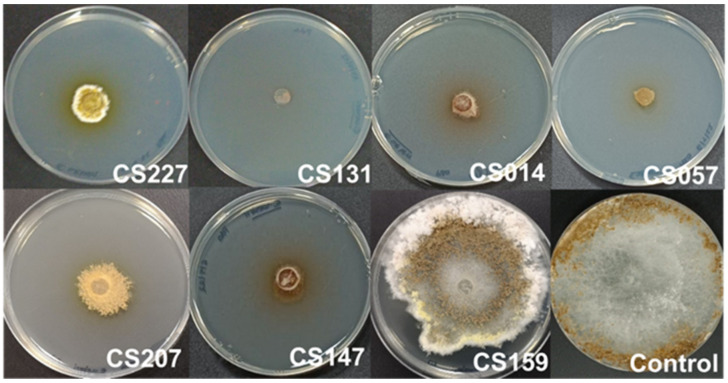
Antifungal effect of *Streptomyces*’s volatile organic compounds (VOCs) on *Escovopsis weberi*. From left to right, *E. weberi* plate when cocultured in VOC chambers against *Streptomyces* spp. CS227, CS131, CS014, CS057 (first row), CS207, CS147, and CS159 (second row) grown on soy flour mannitol (SFM) medium. As a control for the assay, the last image corresponds to a VOC chamber without a *Streptomyces* sp. culture.

Specifically, in this test, we observed how certain *Streptomyces* strains growing on SFM medium inhibited or delayed the growth of the pathogen in comparison with the control sample, which was a plate of *E. weberi* in a VOC chamber without *Streptomyces* spp. Thus, *Streptomyces* spp. CS227, CS014, CS207, CS147, and CS159 delayed *E. weberi*’s growth at different levels, whereas CS131 and CS057 nearly inhibited its development (Figure 2). On the other hand, none of these strains produced antifungal compounds in a suitable quantity to make the effect visible when cultured on R5A. *E. weberi* is a fungus with very large aerial growth, which quickly invades all the available space after a few days of cultivation.

**Figure 3:**
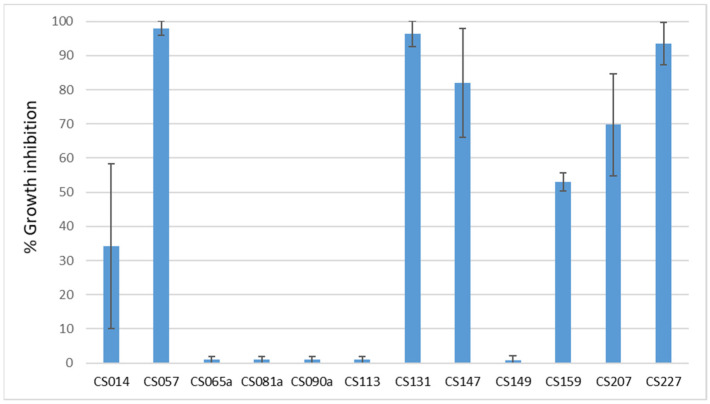
Average growth inhibition of *Escovopsis weberi* (*n* = 3) when cocultured with different *Streptomyces* sp. strains. Strains CS057, CS131, and CS227 cause inhibition above 90%. The standard deviation of the results is represented.

Based on these results, the inhibition percentage (Figure 3) was calculated by measuring the diameter of the colony. It can be seen that strains CS057, CS131, and CS227 cause growth inhibition in *E. weberi* above 90%.

This experiment is the first time that these chambers have been used for bacteria–fungus interaction tests since they were first employed for fungus–fungus [15], fungus–insect [27], fungus–plant [28], and bacteria–bacteria assays [16].

### 3.3. Capture of VOCs and GC-MS Analysis

The capture of volatiles emitted by the coculture was subsequently performed using activated charcoal. The compounds retained in activated charcoal were extracted with ethyl acetate and analyzed via GS-MS. After we positively identified the detected VOCs by comparing their MS spectra with those deposited in the NIST20 library, a clustering study was performed using the R software. In this assay, the Upset Plot is a data visualization method that shows, on a vertical axis, the number of compounds that are exclusively in each sample, the ones that are present in the various samples, and indicating in which. On the horizontal axis, the number of compounds identified in each sample is represented.

This assay was performed with four *Streptomyces* sp. strains (CS057, CS147, CS014, and CS131) on SFM and R5A media. Since the production of antifungal compounds does not occur when the strains are cultivated on R5A (according to Results Section 3.2), all compounds detected in common with the samples that are cultured in SFM are considered not responsible for the antifungal activity.

#### 3.3.1. Cocultured *E. weberi–Streptomyces* sp. CS057

**Figure 4:**
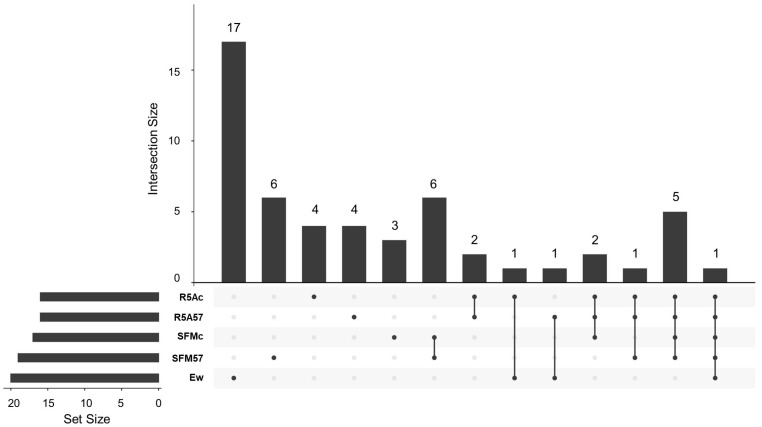
Upset Plot from the compounds detected via GS-MS from the assay confronting *Streptomyces* sp. CS057 and *Escovopsis weberi*. Ew: volatiles produced by *E. weberi*; SFMc: volatile produced during the coculture of *E. weberi*–*Streptomyces* sp. CS057 on soy flour mannitol (SFM) medium; SFM057: volatiles produced by CS57 on SFM; R5A57: volatiles produced by CS057 on R5A medium; R5Ac: volatiles produced during the coculture of *E. weberi*–*Streptomyces* sp. CS057 on R5A.

A total of 53 VOCs from those captured by activated charcoal and extracted with ethyl acetate were positively identified using the NIST20 library (Table S1, Supplementary Material), of which 40 of them were detected in the samples with *Streptomyces* sp. CS057 or its coculture with *E. weberi*. The Upset Plot (Figure 4) showed a wide variety of compounds known to be part of plant essential oils, most of them with bioactivities described in [29,30,31]. Nevertheless, most of these properties are described in essential oils, not in the isolated compounds that comprise them. For instance, calamenene is a component of several plant essential oils with antimicrobial, antiviral, anti-biofilm, and antioxidant activities [32,33,34,35]. S-methyl methanethiosulfonate and allyl nonanoate are related to significant antifungal and antibiotic activity, respectively [36,37]. Sabinene (Bicyclo[3.1.0]hexane, 4-methylene-1-(1-methylethyl)-) is also part of plant oils and has a wide number of applications as flavorings, perfume additives, and biofuels [38]. Similarly, pyrazines have different antimicrobial properties in various applications in the food industry, agriculture, and pharmacy [39,40,41]. Special mention should be made of cubenene o 1,2,3,5,6,8a-hexahydro-4,7-dimethyl-1-(1-methylethyl)γ-cadinene((1S-cis)-Naphthalene, which has strong antitumoral activity against ovarian cancer [42] and antimicrobial bioactivity against different clinical pathogens [32,43], and this compound is identified in various samples in both media and in other strains. The other compounds identified were 2-methylisoborneol or trans-1,10-Dimethyl-trans-9-decalinol (commonly known as geosmin), typically produced by *Streptomyces* [44]. Among these VOCs, only three were exclusively produced and detected in confrontation samples on SFM (Table S2, Supplementary Material).

- **Camphene**: (2,2-dimethyl-3-methylidenebicyclo [2.2.1] heptane). This is a bicyclic terpene produced by various medicinal plants and associated with antibacterial, antifungal, antioxidant, anticancer, antiparasitic, antiviral, anti-inflammatory, and hypolipidemic activities [45]. The remarkable antifungal activity of this compound and its derivatives against diverse pathogenic fungi (*Candida albicans*, *Aspergillus flavus*, *Microsporum canis*, among others) [26,46] supports the hypothesis regarding the activity of this compound, which was exclusively present in the confrontation where antifungal activity was spotted.
- **4-methyl-1-(1-methylethyl)-Bicyclo[3.1.0]hex-2-ene**: (beta-thujene). This terpene has not been found to be produced by bacteria. It has been described as a plant metabolite associated with antifungal and antibacterial activity [47,48]. Despite the high percentage of reliability with which this compound has been identified (>90% in each replicate), we cannot assure the identification of this compound since no bibliographical information supports the bacterial production of beta-thujene. It is remarkable that other similar compounds (4-methylene-1-(1-methylethyl)-bicyclo[3.1.0]hexane and 6-hydroxy-5-methyl-6-vinyl-bicyclo[3.2.0]heptan-2-one) were detected in other samples, so we suggest that perhaps they are various modifications of the same compound caused by interactions with other volatiles that may contain this antifungal activity.
- **2-propenyl ester-octanoic acid**: Acids of this type are lipid residues from alcoholic fermentation that can be subsequently used as a substrate to produce other compounds. Oxalic acid has been reported to exert antifungal activity at certain levels by membrane disruption [49,50]. Although other fatty acids are present in these samples, octanoic acid is only present in SFM confrontation samples in a large enough quantity to be detected using the method followed, so it can be a degradation product that contributes to the VOC chamber environment conditions that affect fungal growth.

Accordingly, it is clear that the presence of *E. weberi* generates a response in *Streptomyces* metabolism. By studying the compounds that were detected via SFM confrontation and SFM-CS057 samples, we identified six compounds shared by both groups (Table S2, Supplementary Material).

2,6,6-Trimethyl (beta-homocyclocitral)1-cyclohexene-1-acetaldehyde [51] and 1,3,3-trimethyl-tricyclo [2.2.1.0(2,6)] heptane (cyclofenche) [52] are compounds with antifungal activities present in essential oils. Similarly, 4,8-dimethylnona-1,3,7-triene is also described as a monoterpene part of plant essential oils with antifungal activities [53]. 3-Octanone has been described as an antifungal cetone [54]. No bioactivity has been described for 3,6-heptanedione and trans 9-methylene-3-oxabicyclo [5.3.0] decan-2-one. These six molecules are common to both groups and contribute to the ones exclusively produced by CS057 on SFM, generating a hostile environment for the growth of *E. weberi*. In summary, the presence of this fungus generates a response in the metabolism of *Streptomyces*, leading to the production of octanoic acid and other VOCs.

In addition, taking into account the biosynthetic gene clusters present in the genome of *Streptomyces* sp. CS057 [5] (predicted using AntiSMASH [55]), of the potential production of terpene-type compounds, there are only two possible pathways responsible for the synthesis of the terpenes detected during this work. On one hand, cluster 1.31 might be responsible for the synthesis of geosmin since it has 100% similarity with the described cluster for the synthesis of this compound. This agrees with results observed at the experimental level since every sample extracted from CS057 cultures (in SFM, R5A, and confrontations against *E. weberi*) showed the presence of related compounds such as trans-1,10-dimethyl-trans-9-decalinol and other molecules with a naphthalene structure. In addition, cluster 1.10 is predicted to be responsible for the synthesis of terpene and has a 69% similarity with the corresponding cluster for hopene biosynthesis. Similarly, cluster 1.11 is predicted to be responsible for 2-methylisoborneol (100% similarity with the described cluster). On the other hand, cluster 1.15 (no similarity with the other described clusters) and cluster 1.26 (19% similarity with the steffimycin D cluster) could be responsible for the biosynthesis of some of the terpenes identified via GS-MS in this strain since terpene synthase/cyclase genes were identified on them (*ctg1_2020* and *ctg1_6135* respectively). Terpenoids are diverse natural compounds obtained from tailoring steps after initial products from terpene synthases, which explains the large number of terpenes identified in comparison with the number of clusters involved in their synthesis [56].

#### 3.3.2. Cocultured *E. weberi*–*Streptomyces* sp. CS131

**Figure 5:**
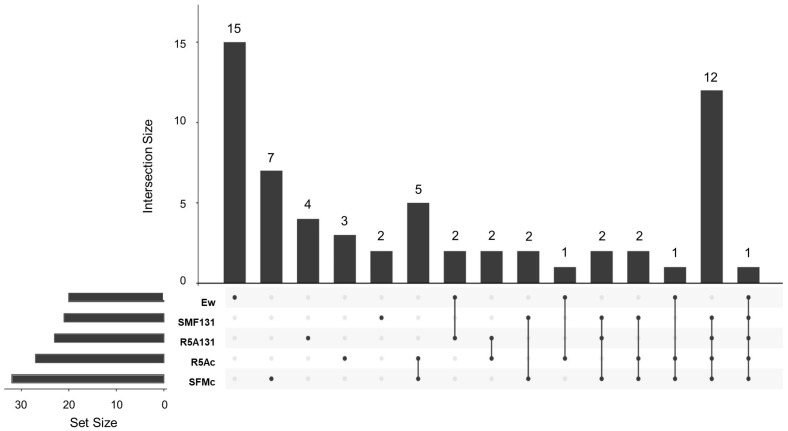
Upset Plot from the compounds detected via GS-MS from the assay confronting *Streptomyces* sp. CS131 and *Escovopsis weberi*. Ew: volatiles produced by *E. weberi*; SFMc: volatile produced during the coculture of *E. weberi–Streptomyces* sp. CS131 on soy flour mannitol (SFM) medium; SFM131: volatiles produced by CS131 on SFM; R5A131: volatiles produced by CS131 on R5A medium; R5Ac: volatiles produced during the coculture of *E.weberi*–*Streptomyces* sp. CS131 on R5A.

The assays confronting strain CS131 with *E. weberi* showed that there were sixty-one compounds captured by the activated carbon and extracted with ethyl acetate, detected via GS-MS and identified using the NIST20 library (Table S3, Supplementary Material). Of these compounds, only seven were exclusively produced and detected in confrontation samples on SFM medium (Table S4, Supplementary Material). The other fifty-four were also produced by other samples (Figure 5). A broad number of properties have been described in the compounds identified. As expected, 2-methylisoborneol and geosmin were detected. Likewise, different compounds with antimicrobial activity were detected, such as furanones, dimethyl disulfide, 2,6,6-trimethyl-1-Cyclohexene-1-acetaldehyde, and muurola derivatives [53,57,58]. It is worth highlighting the production of caryophyllenyl alcohol, identified in several samples in both media and associated with several applications in cosmetics and derivatives in the drug industry (hypolipidemic, anti-inflammatory properties [59,60,61,62].

Those compounds that were exclusively produced via SFM medium confrontation demanded attention: no activity has been described for 1,2,4,5-Tetrazin-3-amine or bis(1,1,3,3-tetramethylbutyl) disulfide in the literature. However, antifungal activity was attributed to 1,2,4,5 tetrazines derivatives [63], some sulfides, and bis(1,1,3,3-tetramethylbutyl) derivatives [58]. 3-octanone and bicyclo derivatives are described in the previous section as antifungal compounds [54,64]. Other VOCs identified in these experiments were

- **Linalool oxide**: This is a monoterpene acyclic tertiary alcohol that has been described as having antifungal activity against the fungus plant pathogen *Guignardia camelliae* [65], *Candida albicans* [66], and *Trichophyton rubrum* [67]. It has a lot of applications as a vitamin E precursor, in cosmetics, and in detergents, being the most widely used terpene in the food industry because of its fragrant and flavor properties [68]. This compound is also produced by many plants (*Coriandrum sativum L.*, *Cymbopogon martini* var 11artini, *Citrus sinensis* Osbeck, among others) as part of their essential oils [69].
- **Cis-5-ethenyltetrahydro-alpha, alpha, 5-trimethyl-2-Furanmethanol (linalool oxide B)**: This is a derivate of the previous molecule, present in the essential oils of several plants of the *Pittosporum* genus (among others), with cytotoxic, antimicrobial, and anti-inflammatory activities [70].
- **2-Methylenebornane**: This is a dehydrated form of 2-methylisoborneol produced by several microorganisms, especially Actinomycetes. This compound, together with geosmin, is responsible for odors in water and soil. It has been reported that several antimicrobial activities are exerted by this compound [65,71].

All these VOCs might be involved in the inhibition of *E. weberi* growth. The analysis of volatile compounds produced both by CS131 confrontation on SFM and CS131 on SFM media (Table S4, Supplementary Material) also reveals the production of compounds with antimicrobial activity, such as trans-linalool oxide, whose activity depends on its stereochemical form [70]. In addition, different tetrasulfides with described antifungal activities [58] were detected.

Based on a bioinformatic analysis of the *Streptomyces* sp. CS131 genome [5], it can be seen that cluster 1.3 might be responsible for geosmin biosynthesis (100% similarity with the previously described cluster). Similarly, clusters 1.7 and 1.33a are involved in isorenieratene biosynthesis (87% and 100% similarity), and cluster 1.30 is involved in hopene biosynthesis (69% similarity). Clusters 1.9 (19% similarity with steffimycin biosynthesis cluster) and 1.23 (no similarity with any cluster described) have a low percentage of similarity with other described clusters, so they could be responsible for the synthesis of some of the terpenes identified using GS-MS in this strain since terpene cyclase/synthase genes have been identified in them (*ctg1_942* and *ctg1_5031*, respectively) and, therefore, in the topic of interest.

#### 3.3.3. Cocultured *E.weberi*–*Streptomyces* sp. CS147

**Figure 6:**
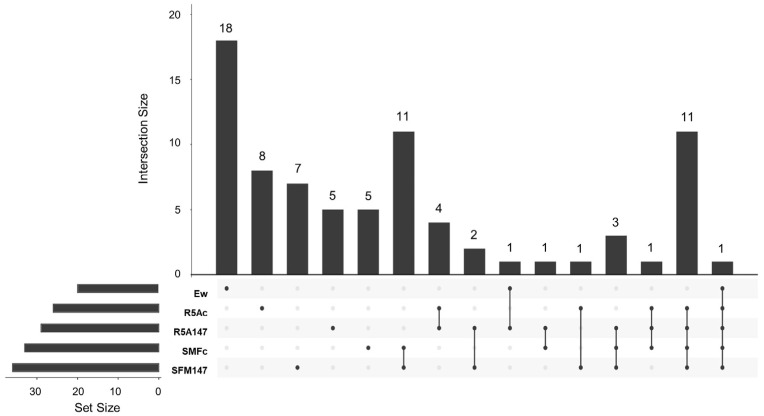
Upset Plot from the compounds detected via GS-MS from the assay confronting *Streptomyces* sp. CS147 and *Escovopsis weberi*. Ew: volatiles produced by *E. weberi*; SFMc: volatile produced during the coculture of *E. weberi–Streptomyces* CS147 on soy flour mannitol (SFM) medium; SFM147: volatiles produced by CS147 on SFM; R5A147: volatiles produced by CS147 on R5A medium; R5Ac: volatiles produced during the coculture of *E. weberi–Streptomyces* sp. CS147 on R5A.

The assays confronting the *Streptomyces* sp. CS147 strain with *E. weberi* on SFM medium showed that a total of seventy-nine compounds were captured by the activated carbon and extracted with ethyl acetate; they were detected via GS-MS and identified using the NIST20 library (Table S5, Supplementary Material). From those, only five were exclusively produced and detected in the confrontation samples on SFM medium (Table S6, Supplementary Material). The other twenty-eight were also produced by other samples (Figure 6).

About these five compounds were found exclusively during SFM medium confrontation, we observed that 3-octanone, previously described as having antifungal properties [54], was also detected in this strain. No antimicrobial activity has been described for 3,5-Di-tert-butyl-2-hydroxybenzaldehyde in the literature. Concerning other VOC identifications, see the following:

- **5-diene-cis-Muurola-4(15)**: The derived compounds are components of plant essential oils that show antimicrobial properties [57,72,73].
- **2-methyl-nonadecane: This** has been described as being produced by *Streptomyces* sp. strains, and it is described as a molecule that contributes to the inhibition of phytopathogenic fungi. *Streptomyces* strain H3-2 has been sought to control Banana Fusarium Wilt because of the production of 2-methylnonadecane, among other volatile compounds [74].
- **1,3-dihydro-5-methoxy-2H-Benzimidazol-2-one:** No bioactivity has been described for this compound, but some derivatives of this molecule are related to antimicrobial activities. Agastache honey, which has 1,3-dihydro-5-methyl 2H-benzimidazol-2-one in its composition, has been described as presenting antifungal properties [75].

The analysis of compounds produced by *Streptomyces* sp. CS147 confrontation on SFM and CS147 on SFM also reveal the production of other compounds with antimicrobial activity: camphene, 1,3,3-trimethyl-tricyclo [2.2.1.0(2,6)] heptane, and 2-methylborname, whose properties have been described above. It is important to highlight the coincidence of many compounds between the assays of the different strains employed in this study. Dimethyl trisulfide and (1S)-6,6-dimethyl-2-methylene-bicyclo [3.1.1] heptane (2(10)-Pinene) have also been described as having antimicrobial activities [76,77]. 1,1,4,4-tetramethyl-2,5-dimethylene-cyclohexane is a volatile produced by *Streptomyces* sp. Y1-14 that has shown antifungal activity against several fungi [77,78]. Azulenone derivatives have been described as part of essential oils with antimicrobial and cytotoxic activities. A similar effect occurs in benzoxepines with antifungal properties [79].

No biological activity has been described for 1,3-bis(1-methylethyl)-1,3-cyclopentadiene, but its properties as an antioxidant and antifungal have been shown in some 1,3-cyclopentadiene derivatives [80]. Neither activity has been described for trans-9-methylene-3-oxabicyclo[5.3.0]decan-2-one.

According to an AntiSMASH [55] analysis of the *Streptomyces* sp. CS147 genome [5], cluster 1.3 is predicted to be involved in geosmin synthesis (100% similarity) and 1.24 in hopene synthesis (69% similarity). Furthermore, cluster 1.23 seems to be implicated in 2-methylisoborneol biosynthesis (100% similarity with the described cluster). On the other hand, clusters 1.7 and 1.26a are predictably involved in isorenieratene biosynthesis (87% and 100% similarity, respectively). In contrast, cluster 1.8 (19% similarity with steffimycin D BGC) and cluster 1.19 (no similarity with any cluster described) show low similarity with the described clusters, but terpene cyclases/synthases have been identified *(ctg1_1003* and *ctg1_5179*, respectively), which suggest their involvement in terpene synthesis.

#### 3.3.4. Cocultured *E. weberi–Streptomyces* sp. CS014

**Figure 7:**
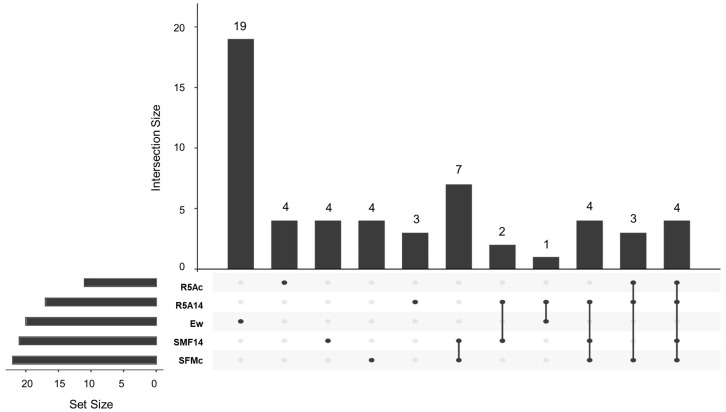
Upset Plot from the compounds detected via GS-MS from the assay confronting *Streptomyces* sp. CS014 and *Escovopsis weberi*. Ew: volatiles produced by *E. weberi*; SFMc: volatile produced during the coculture of *E. weberi–Streptomyces* sp. CS014 on soy flour mannitol (SFM) medium; SFM14: volatiles produced by CS014 on SFM; R5A14: volatiles produced by CS014 on R5A medium; R5Ac: volatiles produced during the coculture *E. weberi–Streptomyces* sp. CS014 on R5A.

Assays confronting strain CS014 with *E. weberi* showed that there were fifty-five volatiles captured by the activated carbon and extracted with ethyl acetate, detected via GS-MS and identified using the NIST20 library (Table S7, Supplementary Material). Of these, only four were exclusively produced and detected in confrontation samples on SFM medium (Table S8, Supplementary Material). The other eighteen were also detected in other samples (Figure 7).

As with the other strains, bibliographic research was performed on the compounds produced exclusively by the confrontation on SFM medium and its properties. 3-octanone and benzoxepin derivatives have been previously mentioned as having antifungal properties [54,79]. 5-methyl dodecane is considered a modified molecule of dodecane and was present in the control samples. The fourth volatile corresponds to alpha-calacorene, a sesquiterpenoid and component of essential oils produced by *Teucrium montanum*, *Daniellia oliveri*, and *Leptoderris micrantha*, among other plants, which has also shown antimicrobial properties [81,82].

The analysis of compounds produced by CS014 and CS014 confrontations on SFM also revealed the production of other compounds with antimicrobial activity: azulene derivatives such as 1,2,3,3a,4,5,6,7-octahydro-1,4-dimethyl-7-(1-methylethenyl), [1R-(1.alpha.,3a.beta.,4.alpha.,7.beta.)]-azulene, and (S)-4,5,6,7,8,8a-hexahydro-8a-methyl-2(1H)-azulenone have been previously mentioned as components of essential oils with antifungal activities. The same applies to liguloxide, which is also part of plant essential oils [83]. (1S,4S,4aS)-1-Isopropyl-4,7-dimethyl-1,2,3,4,4a,5-hexahydronaphthalene is a stereoisomer of cis-muurola-3,5-diene, which has already been described [59]. Previously, we revealed the antifungal properties of disulfite and furanone derivatives. However, no bioactivity has been described for 2-ethyl-heptane.

According to an AntiSMASH analysis of the *Streptomyces* sp. CS014 genome [5] regarding the identification of terpene biosynthesis gene clusters, cluster 1.4 is predicted to be involved in hopene synthesis (69% similarity), 1.20 is involved in isorenieratene biosynthesis (19% similarity), and 1.23 is involved in geosmin production (100% similarity). On the other hand, cluster 1.10 is also supposedly involved in the biosynthesis of terpene compounds because it has no similarity with any described cluster and has a terpene synthase/cyclase gene (*ctg1_1705*). This is the same for cluster 1.19 (19% similarity with steffimycin), which has the *ctg1_5927* gene.

It is worth noting that, of the four strains employed in this study, the predictions provided by AntiSMASH for terpene biosynthesis were very similar, which suggests similarities between microorganisms isolated from the same niche.

The analysis methodology used in this work to deal with VOCs allows for the extraction of the large number of volatile compounds retained in activated carbon [84]. However, it is important to note that this method has some limitations. On the one hand, there might be volatile compounds that are not retained in the activated charcoal, or they might volatilize when the recovery matrix is collected. Also, when the compounds are extracted with ethyl acetate, we can delimitate the analysis exclusively to those dissolved with that solvent. In addition, the GS-MS methodology presents a limit of detection in such a way that those compounds that present at low concentrations are not properly detected. Finally, those that were not registered in the referenced library or that have not been described could not be identified and, consequently, they were discarded from the study. Thus, further analysis will be carried out to expand these studies.

It is worth mentioning that the numerous compounds that the strains employed produce are described as being part of the essential oils of different plants and used for different medicinal properties. Just as *Streptomyces* spp. produces various bioactive compounds that are also produced by plants [85], it makes sense that this phenomenon also occurs in volatile compounds.

All the results presented above suggest that various compounds might be responsible for the observed antifungal activity, mainly because of the abundant different VOCs identified in each analytical sample. It is important to highlight that the present work is not intended to assign antifungal activity to a single compound. The origin of a certain activity is usually linked to a particular compound or various compounds [86], not always in a causal relationship of 1:1. In these experiments, it can be seen how several compounds could jointly contribute to generating an environment hostile to fungal growth. The additive or synergistic effect between different volatiles results in a delay or inhibition of fungal growth. It seems straightforward that, for strains showing strong activity against *E. weberi*, antifungal compounds might normally be produced to a lesser extent, while a confrontation with *E. weberi* stimulates or increases the production of compounds that contribute to generating adverse conditions for fungal growth. It must also be taken into account that many compounds, such as (1S,4,4aS)-1-Isopropyl-4,7-dimethyl-1,2,3,4,4a,5-hexahydronaphthalene in CS131, are not only found in samples of interest generated on SFM medium but are also produced by other strains grown on R5A medium. This means that such VOCs cannot be held responsible for antifungal activity, but neither can their collaboration in the final antifungal activity be denied. Despite all the descriptions of antifungal compounds made above, many of them have not been described individually as possessing these properties, but most of them are part of essential oil mixtures with antifungal activities without knowing if each one of their individual components has that particular effect, so it cannot be denied that some of them may have the aforementioned activity in and of themselves.

### 3.4. Dual Cultures against Other Fungi

In order to evaluate the antifungal effect on other fungi that are not pathogenic in the natural environment in which these *Streptomyces* spp. were isolated, an experiment was performed employing VOC chambers and using *Sclerotinia* spp. and *Phytophthora cinnamomi.* Both fungi are highly destructive plant pathogens [87,88]. *Sclerotinia* spp., like *E. weberi*, belong to the Ascomycota phylum, so their characteristics and life cycle are quite similar. Conversely, *P. cinnamomi* is an Oomycete, which implies more differences from the *E. weberi* pathogen.

**Figure 8:**
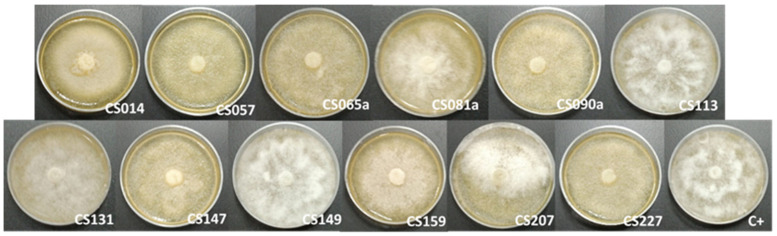
*Phytophthora cinnamomi* plates when cocultured in volatile organic compound (VOC) chambers against *Streptomyces* spp. CS014, CS57, CS065a, CS081a, CS090a, CS113 (first row), CS131, CS147, CS149, CS159, CS207, and CS227 (second row). The last image corresponds to the control of the assay (a VOC chamber without a *Streptomyces* sp. culture).

**Figure 9:**
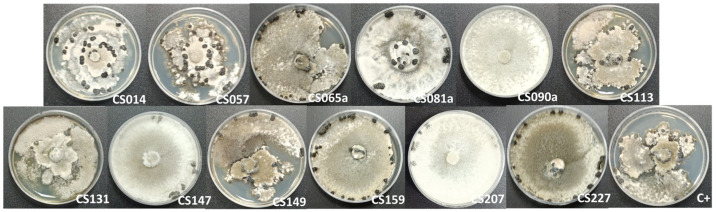
*Sclerotinia* spp. plates when cocultured in VOC (volatile organic compound) chambers against *Streptomyces* sp. CS014, CS57, CS065a, CS081a, CS090a, CS113 (first row), CS131, CS147, CS149, CS159, CS207, and CS227 (second row). The last image corresponds to the control of the assay (a VOC chamber without a *Streptomyces* sp. culture).

The results showed that all of the *Streptomyces* sp. strains tested did not present antifungal activity against either of the two fungi (Figure 8 and Figure 9). This fact represented an intriguing outcome since the production of volatile antifungal compounds by different *Streptomyces* strains is clearly not enough to prevent fungal growth in general terms. In addition, the results pointed to ecological implications supported by particular components that determine the observed effect manifesting solely against *E. weberi*, which is the natural pathogen in the environment where these *Streptomyces* strains were isolated. We cannot assume that the effect is exclusive and specific to *E. weberi*, but, at least, it is not effective on all types of fungi. Dhodary et al. [84] pointed out that this response could be a result of ammonia production by the different strains because *E. weberi* is highly sensitive to basification.

### 3.5. Analysis of Diffusible Compound Production

Culture samples of the different strains that showed antifungal effects on *E. weberi* were extracted with different organic solvents. This assay aimed to evaluate whether there were differences in the chromatographic profile of the *Streptomyces* spp. confronted with *E. weberi* in comparison with their counterparts confronted with *P. cinnamomi* (where no antifungal activity was detected) and those not confronted with any microorganism.

The results obtained showed that, in the conditions tested, there are some differences between the diffusible production profiles of *Streptomyces* spp. when confronted with *E. weberi* or *P. cinnamomi* and when there is no confrontation. In the case of strain CS057, the activation of skyllamycins A and B can be seen. In addition, in the CS014 activation of different granaticins, it can also be seen. Therefore, in terms of diffusible compound production, it is clear that the *Streptomyces* strains we tested react similarly to the near presence of both *E. weberi* and *P. cinnamomi* (Figure S3, Supplementary Material).

### 3.6. Multiple Culture

To evaluate if there is any synergistic antifungal effect when different *Streptomyces* spp. are present at the same time in a confined environment in the presence of *E. weberi*, three representatives of those microorganisms were cultured in small open dishes inside a large dish. In this way, we simulated an environment similar to that of a VOC chamber, where there is no physical contact between microorganisms or their diffusible compounds but there is an exchange of volatiles.

**Figure 10:**
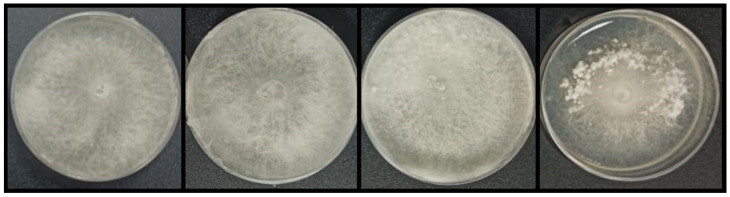
Reduction in *Escovopsis weberi* growth via multiple cocultures of different *Streptomyces* sp. strains. From left to right, *E. weberi* when cultured alone; *E. weberi* cultured in the presence of strain CS081a; *E. weberi* cultured in the presence of strain CS090a; *E. weberi* cultured in the presence of strains CS081a and CS090a.

The results showed that certain combinations of isolated *Streptomyces* spp. increased their antifungal activity against *E. weberi,* a sum effect not observed with each individual strain alone. This effect is probably due to the saturation of antimicrobial compounds in the environment. Even though some *Streptomyces* spp. did not show antifungal activity in those assays that were previously shown (most probably because they do not produce VOCs in a large enough quantity or because they produce few compounds with the required activity by accumulating both volatile productions together), it seems obvious that the joint effect provokes a manifest reduction in *E. weberi* growth (Figure 10).

### 3.7. pH Variation Analysis

It has been noted that the growth of *E. weberi* is inhibited by ammonia production, while *Leucoagaricus gongylophorus,* a fungus that lives in the environment of leafcutter ants, is not [84]. The role of this last fungus is very important for the ant ecosystem since it is in charge of degrading plant matter, thus serving as food for the ants [89].

**Figure 11:**
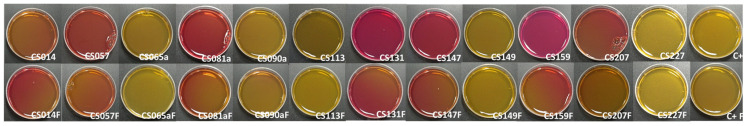
Potato Dextrose Agar (PDA) + phenol red plates in VOC (volatile organic compound) chambers in the presence of *Streptomyces* spp. First row corresponds to plates cocultured with *Streptomyces* spp.; second row corresponds to plates cocultured with *Streptomyces* spp. and *Escovopsis weberi*.

To evaluate if the basification of the environment caused by volatile compounds can contribute to the inhibition of *E. weberi*, an assay with three small open plates inside a large one was performed: a plate containing *E. weberi*, a plate harboring a *Streptomyces* strain, and a plate of PDA supplemented with phenol red as pH indicator. Phenol red reagent is yellow at a pH below 6.8 and turns red when it is over 7.4. The results showed that all the strains that reveal antifungal activity against *E. weberi* make the indicator plate turn red at different levels, except for strain CS227 (Figure 11), whose color change is very slight.

These results suggest that the basification of an environment can contribute, together with the antifungal compounds previously described, to generating a hostile environment for pathogen development. In the case of CS227, it probably does not produce ammonia in a large enough quantity to provoke changes in the medium’s pH. However, we do not assume that the inhibition is just induced by environment basification, but it might be one additional element that participates in the antifungal conditions generated. Supporting the previous assertion, strain CS081a has no volatile bioactivity against *E. weberi,* but it induces volatile basification in PDA plates (Figure 11). This fact highlights our hypothesis that the environmental basification of a medium can influence the antifungal bioactivity of these strains, but this is not the only factor.

It has been noted that in cocultures of *Streptomyces* spp. and yeast, once the nutrient source is depleted, the *Streptomyces* spp. are stimulated to produce trimethylamine (TMA), which not only alkalizes the environment but also favors its exploratory potential [6]. In the fight for space, nutrients, the colonization of an environment, and the chance to parasitize other organisms, microorganisms use all kinds of strategies to make their way into the ecosystem, thus inducing a wide variety of responses from other competitors. These tools are essential for the correct maintenance of the environment, and their absence might cause an imbalance with a consequent domino effect on other members of that ecological niche or trophic chain.

We have shown that a combination of different elements with various natures and properties collaborate to inhibit pathogen development. It has previously been noted that volatile compounds can impede or slow entomopathogenic growth [90,91]. Although there are multiple reasons why bacteria produce volatile compounds (signaling, competition, etc.), it is plausible that the production of antifungal compounds could be because bacteria grow slower than fungi.

The present work shows the importance of VOCs in soil communities where different organisms might be dwelling in close proximity but at distances where the diffusible antimicrobial compounds do not have an action range [5]. The study of VOC emissions, typically performed using GS-MS, is difficult given the gaseous nature of the compounds and the ease of sample contamination from other volatiles present in the environment. In addition, the culture media not only determines the bacterial production of diffusible compounds but also produces volatile compounds [90,92]. However, with those limitations, it has been estimated that nearly 50–80% of bacteria produce volatile compounds under laboratory conditions [90], so it is worth focusing efforts on the discovery of new VOCs with recognized biological activities.

## 4. Conclusions

From the analysis of the antifungal activity of the twelve *Streptomyces* strains isolated from leafcutter ants, we found that seven of them showed antifungal activity against *E. weberi,* causing inhibition or a great delay in the fungal growth. The volatilomes of four of these strains were analyzed via GS-MS, revealing the presence of a large number of compounds, many of them described in the bibliography as antifungal or belonging to mixtures exerting such activity. These findings suggest that this inhibition could be due to the accumulation of various compounds with antimicrobial properties, excluding the option of assigning the observed activity to a specific compound. On the other hand, it has been estimated that the ammonium produced by these strains also contributes to generating an unfavorable environment for *E. weberi* growth, although that factor is not the only reason for the antifungal activity. In addition, this work reveals, once again, the multitude and complexity of the interactions that take place in ant nests and the bioactive potential of microorganisms isolated from this environment.

## Data Availability

Not applicable.

## Supplementary Materials

The following supporting information can be downloaded at https://www.mdpi.com/article/10.3390/microorganisms11071820/s1: Figure S1: Device used on multiple cocultures of *Escovopsis weberi*–*Streptomyces* spp. Figure S2: Coculture of *Escovopsis weberi* and *Streptomyces* spp. on PDA, R5A, and SFM. Figure S3: Overview of chromatographic profile of *Streptomyces* sp. CS057 when cultured on SFM and extracted with butanol. Table S1: Compounds identified via GS-MS in the assay of volatile organic compounds produced by the confrontation of *Streptomyces* sp. CS057 and *E. weberi*. Table S2: Summary of compounds identified via GS-MS in the assay of volatile organic compounds produced by the confrontation of *Streptomyces* sp. CS057 and *E. weberi.* Table S3: Compounds identified via GS-MS in the assay of volatile organic compounds produced by the confrontation of *Streptomyces* sp. CS131 and *E. weberi.* Table S4: Summary of compounds identified via GS-MS in the assay of volatile organic compounds produced by the confrontation of *Streptomyces* sp. CS131 and *E. weberi.* Table S5: Compounds identified via GS-MS in the assay of volatile organic compounds produced by the confrontation of *Streptomyces* sp. CS147 and *E. weberi*. Table S6: Summary of compounds identified via GS-MS in the assay of volatile organic compounds produced by the confrontation of *Streptomyces* sp. CS147 and *E. weberi.* Table S7: Compounds identified via GS-MS in the assay of volatile organic compounds produced by the confrontation of *Streptomyces* sp. CS014 and *E. weberi*. Table S8: Summary of compounds identified via GS-MS in the assay of volatile organic compounds produced by the confrontation of *Streptomyces* sp. CS014 and *E. weberi.*
