# Supplementary material for: The Volatile Organic Compounds of Streptomyces spp.: An In-Depth Analysis of Their Antifungal Properties
Source: Microorganisms. 2023 Jul 16;11(7):1820. doi: 10.3390/microorganisms11071820 (PMC10384482; doi:10.3390/microorganisms11071820)
Supplement: Supplementary file 1 [file microorganisms-11-01820-s001.zip › microorganisms-2501217-supplementary.pdf]

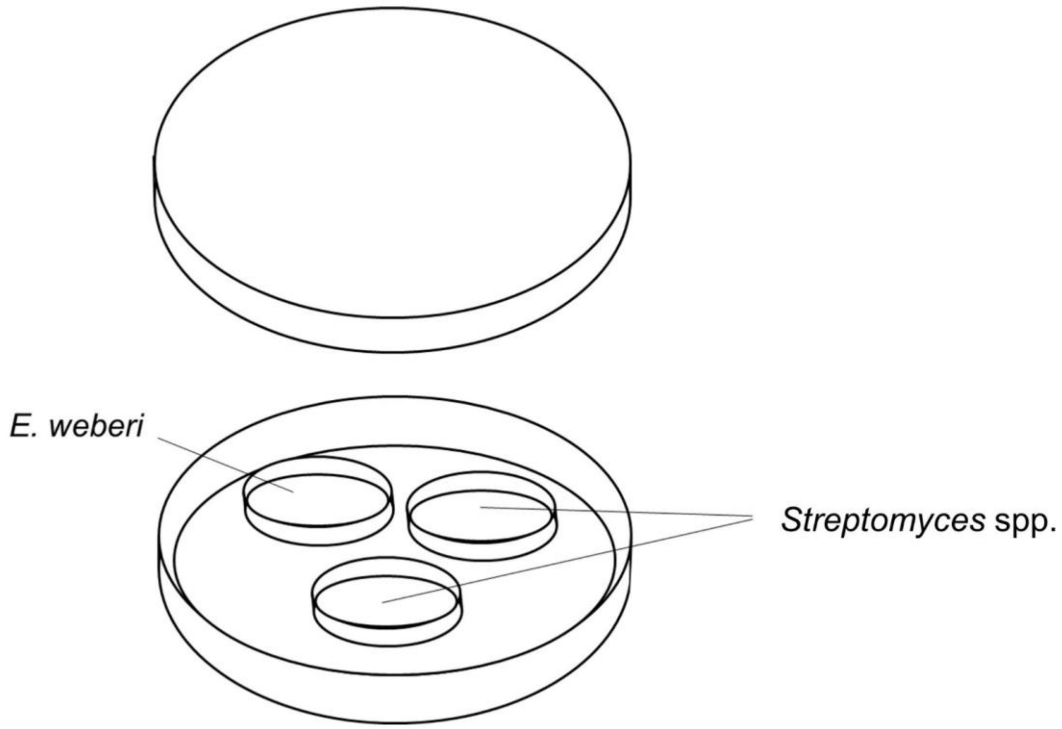

Figure S1: Device used for multiple coculture *Escovopsis weberi-Streptomyces* spp.

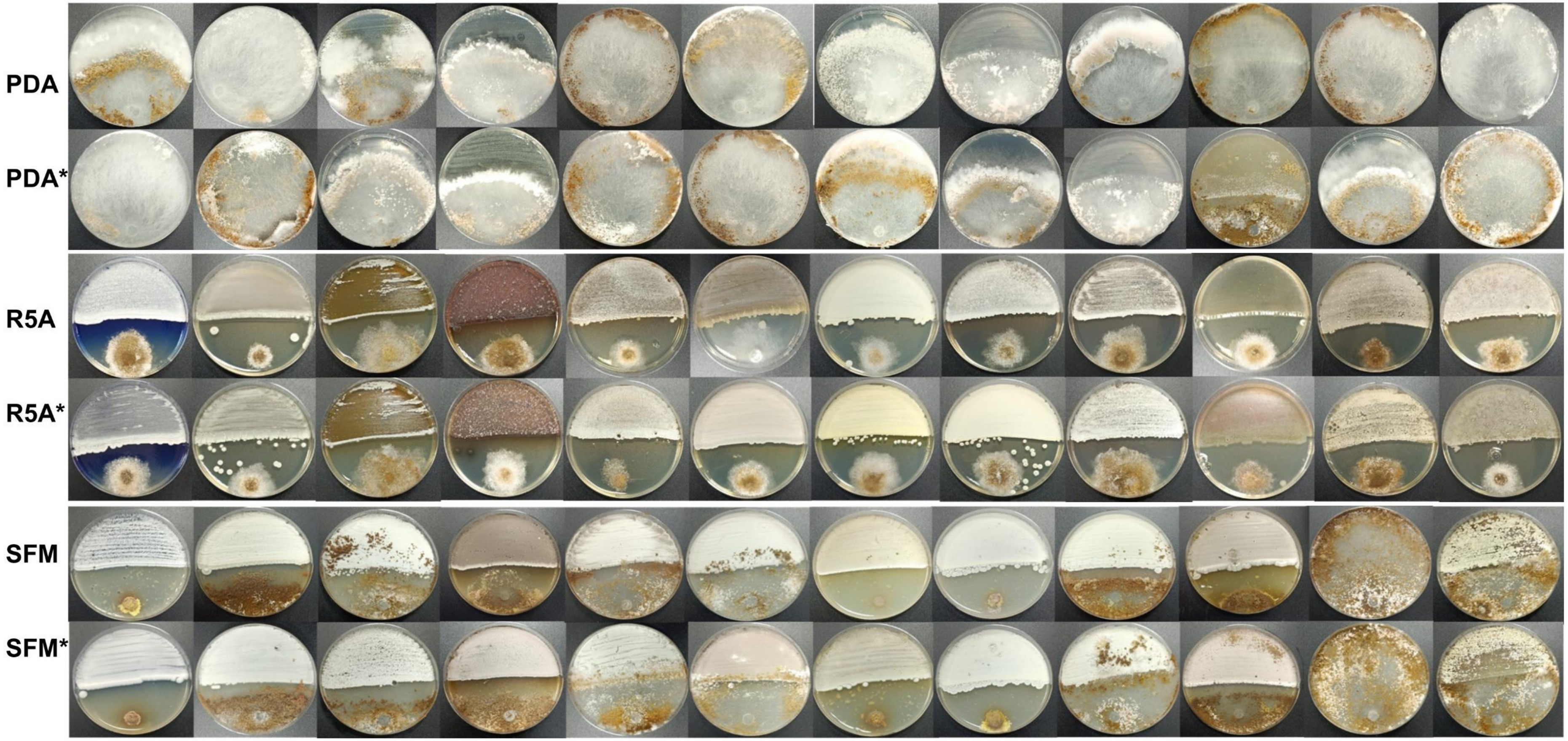

Figure S2. Coculture of *Escovopsis weberi* and *Streptomyces* spp. on Potato Dextrose Agar (PDA), R5A, and Soy Flour Mannitol (SFM) media. The asterisk indicates the confrontation experiment using two days-grown *Streptomyces* spp.

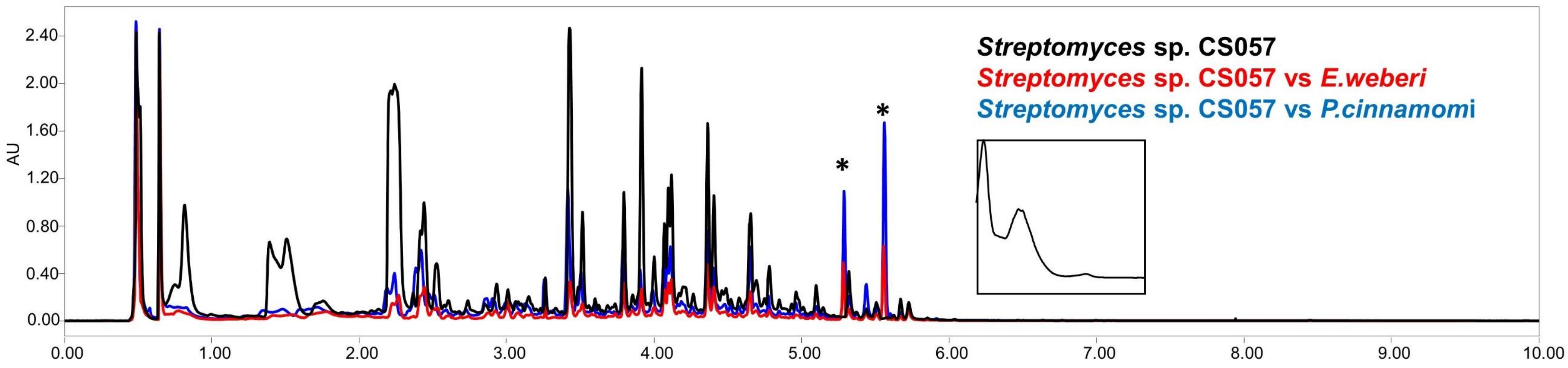

Figure S3. Overview of chromatographic profile of *Streptomyces* sp. CS057 cultured in Soy Flour Mannitol (SFM) medium and extracted with butanol. Activation of skyllamycins A and B biosynthesis can be observed (asterisks) by the presence of fungi *Escovopsis weberi* or *Phytophthora cinnamomi*. UV-Vis spectrum of skyllamycins is shown.

Table S1. Compounds identified by GS-MS in the assay of Volatile Organic Compounds produced by the confrontation *Streptomyces* CS057 and *E. weberi* (Ew: volatiles produced by *E. weberi*; SFM-confrontation: volatiles produced during the confrontation *E.weberi* - *Streptomyces* CS057 on SFM; SFM-CS057: volatiles produced by CS57 on SFM; R5A-CS057: volatiles produced by CS057 on R5A; R5A-confrontation: volatiles produced during the confrontation *E.weberi* - *Streptomyces* CS057).

SFM-CS057	SFM-confrontation	R5A-CS057	R5A-confrontation	Ew
• Naphthalene, 1,2,3,4-tetrahydro-1,6-dimethyl-4-(1-methylethyl)-, (1S-cis)<br>• 1H-Cyclopropa[a]naphthalene, 1a,2,3,5,6,7,7a,7b-octahydro-1,1,7,7a-tetramethyl-, [1aR-(1a.alpha.,7.alpha.,7a.alpha.,7b.alpha.)]<br>• trans-1,10-Dimethyl-trans-9-decalinol<br>• 1-Cyclohexene-1-acetaldehyde, 2,6,6-trimethyl<br>• S-Methylmethanethiosulphonate<br>• p-Menth-8-ene, 3-methylene-<br>• 1,3-Cyclopentadiene, 1,3-bis(1-methylethyl)-	• Tricyclo[2.2.1.0(2,6)]heptane, 1,3,3-trimethyl<br>• Camphene<br>• Dimethyltrisulfide<br>• Bicyclo[3.1.0]hex-2-ene, 4-methyl-1-(1-methylethyl)-<br>• 3-Octanone<br>• p-Menth-8-ene, 3-methylene<br>• 3,6-Heptanedione<br>• Octanoic acid, 2-propenyl ester<br>• 4,8-dimethylnona-1,3,7-triene<br>• 2,3-Dimethyl-5-oxohexanethioic acid, S-t-butylester<br>• 3-Oxabicyclo[5.3.0]decan-2-one, 9-methylene-, trans	• Pyrazine, 2,5-dimethyl-<br>• Dimethyltrisulfide<br>• Benzene, 1-ethoxy-4-ethyl-<br>• p-Menth-8-ene, 3-methylene-<br>• S-Methylmethanethiosulphonate<br>• Vinyl 2-ethylhexanoate<br>• 2,3-Dimethyl-5-oxohexanethioic acid, S-t-butylester<br>• Bromoacetylbromide<br>• 2-Methylisoborneol<br>• Allylnonanoate<br>• trans-1,10-Dimethyl-trans-9-decalinol	• 3,5-Di-tert-butyl-2-hydroxybenzaldehyde<br>• cis-Calamenene<br>• Naphthalene, 1,2,3,5,6,8a-hexahydro-4,7-dimethyl-1-(1-methylethyl)-, (1S-cis)<br>• D-Alanine, N-(4-butylbenzoyl)-, heptylester<br>• 1H-Cyclopropa[a]naphthalene, 1a,2,3,5,6,7,7a,7b-octahydro--1,1,7,7a-tetramethyl-, [1aR-(1a.alpha.,7.alpha.,7a.alpha.,7b.alpha.)]-<br>• trans-1,10-Dimethyl-trans-9-decalinol<br>• 2,4,4,6,6,8,8-Heptamethyl-2-nonene	• 2,6-Di-tert-butyl-4-hydroxy-4-methylcyclohexa-2,5-dien-1-one<br>• Isophthalicacid, 3,5-difluorophenyl heptylester<br>• Phosphoramidousdifluoride, dimethyl<br>• 3-Butene-1,2-diol<br>• 1-Dodecanol<br>• Bicyclo[3.2.0]heptan-2-one, 6-hydroxy-5-methyl-6-vinyl<br>• Succinimide<br>• Pentanoicacid, 5-hydroxy-, 2,4-di-t-butylphenyl esters<br>• 2-Methylisoborneol<br>• Heptadecane

Table S1. Cont.

SFM-CS057	SFM-confrontation	R5A-CS057	R5A-confrontation	Ew
• Bicyclo[3.1.0]hexane, 4-methylene-1-(1-methylethyl)-<br>• Dimethyltrisulfide<br>• 2,3-Dimethyl-5-oxohexanethioic acid, S-t-butylester<br>• Tricyclo[2.2.1.0(2,6)]heptane, 1,3,3-trimethyl<br>• 3-Octanone<br>• 3,6-Heptanedione<br>• 4,8-dimethylnona-1,3,7-triene<br>• 3-Oxabicyclo[5.3.0]decan-2-one, 9-methylene-, trans<br>• Aceticacid, trifloro-,1-methylpropyl ester<br>• 2-Methylisoborneol	• 2-Methylisoborneol<br>• 1-Cyclohexene-1-acetaldehyde, 2,6,6-trimethyl<br>• trans-1,10-Dimethyl-trans-9-decalinol<br>• 1H-Cyclopropa[a]naphthalene, 1a,2,3,5,6,7,7a,7b-octahydro-1,1,7,7a-tetramethyl-, [1aR-(1a.alpha.,7.alpha.,7a.alpha.,7b.alpha.)]<br>• Naphthalene, 1,2,3,5,6,8a-hexahydro-4,7-dimethyl-1-(1-methylethyl)-, (1S-cis)<br>• cis-Calamenene	• 1H-Cyclopropa[a]naphthalene, -<br>• 1a,2,3,5,6,7,7a,7b-octahydro-1,1,7,7a-tetramethyl-, [1aR-(1a.alpha.,7.alpha.,7a.alpha.,7b.alpha.)]<br>• Bicyclosesquiphellandrene<br>• Naphthalene, 1,2,3,5,6,8a-hexahydro-4,7-dimethyl-1-(1-methylethyl)-, (1S-cis)-<br>• cis-Calamenene<br>• Tris(3-phenyl-2,4-pentanedionato)aluminum(iii)	• 1,5-Dioxaspiro[5.5]undecane, 3,3-dimethyl<br>• Allylnonanoate<br>• 2-Methylisoborneol<br>• 2,3-Dimethyl-5-oxohexanethioic acid, S-t-butylester<br>• S-Methylmethanethiosulphonate<br>• p-Menth-8-ene, 3-methylene<br>• Benzene, 1-ethoxy-4-ethyl<br>• Dimethyltrisulfide	• 2,4,4,6,6,8,8-Heptamethyl-2-nonene<br>• Pyrrolidine<br>• Pyrazine, 2,5-dimethyl<br>• Acetophenone<br>• 2-Pentene, 2,4,4-trimethyl<br>• 3-Heptene, 2,2,4,6,6-pentamethyl<br>• Cyclohexane, 1-ethyl-1-methyl<br>• Hexane, 3,3-dimethyl<br>• Bis(2-isopropyl-5-methylcyclohexyl) methylphosphonate (isomer 2)<br>• 3,6-Dimethylpiperazine-2,5-dione

Table S2. Summary of compounds identified by GS-MS in the assay of Volatile Organic Compounds produced by the confrontation of *Streptomyces* CS057 and *E. weberi*. In the first column: VOCs exclusively detected on SFM confrontation; in the second, VOCs in common between SFM confrontation and SFM57.

**VOCs exclusively in CS057 SFM-confrontation**	**VOCs in common in SFM-confrontation and SFM-CS057**
• Camphene<br>• Bicyclo[3.1.0]hex-2-ene, 4-methyl-1-(1-methylethyl)-<br>• Octanoic acid, 2-propenyl ester	• 1-Cyclohexene-1-acetaldehyde, 2,6,6-trimethyl<br>• Tricyclo[2.2.1.0(2,6)]heptane, 1,3,3-trimethyl<br>• 3-Octanone<br>• 3,6-Heptanedione<br>• 4,8-dimethylnona-1,3,7-triene<br>• 3-Oxabicyclo[5.3.0]decan-2-one, 9-methylene-, trans

Table S3. Compounds identified by GS-MS in the assay of Volatile Organic Compounds produced by the confrontation *Streptomyces* CS131 and *E. weberi* (Ew: volatiles produced by *E. weberi*; SFM-confrontation: volatiles produced during the confrontation *E.weberi* - *Streptomyces* CS131 on SFM; SFM-CS131: volatiles produced by CS131 on SFM; R5A-CS131: volatiles produced by CS131 on R5A; R5A-confrontation: volatiles produced during the confrontation *E.weberi* - *Streptomyces* CS131.

SFM-CS131	SFM-confrontation	R5A-CS131	R5A-confrontation	Ew
• 2(3H)-Furanone, dihydro-3-methyl-	• Bicyclo[3.1.0]hex-2-ene, 2-methyl-5-(1-methylethyl)-	• Pyrazine, 2,5-dimethyl-	• 2(3H)-Furanone, dihydro-3-methyl-	• 2,6-Di-tert-butyl-4-hydroxy-4-methylcyclohexa-2,5-dien-1-one
• Dimethyltrisulfide	• 2(3H)-Furanone, dihydro-3-methyl-	• 2(3H)-Furanone, dihydro-3-methyl-	• 1,3-Cyclopentadiene, 1,3-bis(1-methylethyl)-	• Isophthalicacid, 3,5-difluorophenyl heptylester
• 1,3-Cyclopentadiene, 1,3-bis(1-methylethyl)-	• Dimethyltrisulfide	• Dimethyltrisulfide	• 2H-Pyran-2-one, tetrahydro-	• Phosphoramidousdifluoride, dimethyl
• p-Menth-8-ene, 3-methylene-	• Bicyclo[3.1.0]hexane, 4-methylene-1-(1-methylethyl)-	• 1,3-Cyclopentadiene, 1,3-bis(1-methylethyl)-	• Dimethyltrisulfide	• 3-Butene-1,2-diol
• trans-Linalool oxide (furanoid)	• 1,3-Cyclopentadiene, 1,3-bis(1-methylethyl)-	• p-Menth-8-ene, 3-methylene-	• Bicyclo[3.1.0]hexane, 4-methylene-1-(1-methylethyl)-	• 1-Dodecanol
• 3-Oxabicyclo[5.3.0]decan-2-one, 9-methylene-, trans-	• 2-Methylenebornane	• 1,7,7-Trimethylbicyclo[2.2.1]hept-5-en-2-one	• 3-Heptene, 2,2,4,6,6-pentamethyl-	• Bicyclo[3.2.0]heptan-2-one, 6-hydroxy-5-methyl-6-vinyl
• 2-Methylisoborneol	• 3-Octanone	• 2-Methylisoborneol	• p-Menth-8-ene, 3-methylene-	
• Tetrasulfide, dimethyl	• 3-Heptene, 2,2,4,6,6-pentamethyl-		• Cyclopentane, 1,1,3,4-tetramethyl-, cis-	

Table S3. Cont.

SFM-CS131	SFM-confrontation	R5A-CS131	R5A-confrontation	Ew
• 2,6-Dimethyl-2-trans-6-octadiene<br>• 1-Cyclohexene-1-acetaldehyde, 2,6,6-trimethyl-<br>• Cyclohexanone, 2,5-dimethyl-2-(1-methylethenyl)-<br>• 2-Undecanone, 6,10-dimethyl-trans-1,10-Dimethyl-trans-9-decalinol<br>• 9-Undecenal, 2,10-dimethyl-<br>• 1H-Cyclopropa[a]naphthalene, 1a,2,3,5,6,7,7a,7b-octahydro-1,1,7,7a-tetramethyl-, [1aR-(1a.alpha.,7.alpha.,7a.alpha.,7b.alpha.)]-	• Disulfide, bis(1,1,3,3-tetramethylbutyl)<br>• p-Menth-8-ene, 3-methylene-<br>• 2-Furanmethanol, 5-ethenyltetrahydro-.alpha.,.alpha.,5-trimethyl-, cis-<br>• trans-Linalool oxide (furanoid)<br>• Pentane, 3,3-diethyl-<br>• 3-Oxabicyclo[5.3.0]decan-2-one, 9-methylene-, trans-<br>• 2-Methylisoborneol<br>• Tetrasulfide, dimethyl<br>• 1-Cyclohexene-1-acetaldehyde, 2,6,6-trimethyl-	• Fumaricacid, decyl 2-methylcyclohex-1-enylmethyl ester<br>• 1-Cyclohexene-1-acetaldehyde, 2,6,6-trimethyl-<br>• Cyclohexanone, 2,5-dimethyl-2-(1-methylethenyl)-1,5-Heptadien-4-ol, 3,3,6-trimethyl-<br>• Cyclopentane, 1,1,3,4-tetramethyl-, cis-<br>• 2-Undecanone, 6,10-dimethyl-<br>• trans-1,10-Dimethyl-trans-9-decalinol<br>• 1H-Cyclopropa[a]naphthalene, 1a,2,3,5,6,7,7a,7b-octahydro-	• Pentane, 3,3-diethyl-<br>• 4,8-dimethylnona-1,3,7-triene<br>• 3-Oxabicyclo[5.3.0]decan-2-one, 9-methylene-, trans-<br>• 2-Methylisoborneol<br>• 1-Cyclohexene-1-acetaldehyde, 2,6,6-trimethyl-<br>• Cyclohexanone, 2,5-dimethyl-2-(1-methylethenyl)-<br>• 2,4,4,6,6,8,8-Heptamethyl-2-nonene<br>• 1,5-Heptadien-4-ol, 3,3,6-trimethyl-	• Succinimide<br>• Pentanoicacid, 5-hydroxy-, 2,4-di-t-butylphenyl esters<br>• 2-Methylisoborneol<br>• Heptadecane<br>• 2,4,4,6,6,8,8-Heptamethyl-2-nonene<br>• Pyrrolidine<br>• Pyrazine, 2,5-dimethyl

Table S3. Cont.

SFM-CS131	SFM-confrontation	R5A-CS131	R5A-confrontation	Ew
• 1-Isopropyl-4,7-dimethyl-1,2,3,4,5,6-hexahydronaphthalene	• Cyclohexanone, 2,5-dimethyl-2-(1-methylethenyl)-	• 1,1,7,7a-tetramethyl-, [1aR-(1a.alpha.,7.alpha.,7a.alpha.,7b.alpha.)]-	• (1S,2E,6E,10R)-3,7,11,11-Tetramethylbicyclo[8.1.0]undeca-2,6-diene	• Acetophenone
• cis-Muurola-4(15),5-diene	• 1,2,4,5-Tetrazin-3-amine	• (1S,4S,4aS)-1-Isopropyl-4,7-dimethyl-1,2,3,4,4a,5-hexahydronaphthalene	• 1H-Cyclopropa[a]naphthalene, 1a,2,3,5,6,7,7a,7b-octahydro-1,1,7,7a-tetramethyl-, [1aR-(1a.alpha.,7.alpha.,7a.alpha.,7b.alpha.)]-	• 2-Pentene, 2,4,4-trimethyl
• cis-Calamenene	• 2-Undecanone, 6,10-dimethyl-	• cis-Muurola-4(15),5-diene	• 2-Undecanone, 6,10-dimethyl-	• 3-Heptene, 2,2,4,6,6-pentamethyl
• Caryophyllenyl alcohol	• trans-1,10-Dimethyl-trans-9-decalinol	• Naphthalene, 1,2,4a,5,6,8a-hexahydro-4,7-dimethyl-1-(1-methylethyl)-, (1.alpha.,4a.alpha.,8a.alpha.)-	• cis-Calamenene	• Cyclohexane, 1-ethyl-1-methyl
• 4a(2H)-Naphthalenol, 1,3,4,5,6,8a-hexahydro-4,7-dimethyl-1-(1-methylethyl)-, (1S,4S,4aS,8aR)-	• (1S,2E,6E,10R)-3,7,11,11-Tetramethylbicyclo[8.1.0]undeca-2,6-diene	• Hexane, 3,3-dimethyl-	• Caryophyllenyl alcohol	• Hexane, 3,3-dimethyl
	• 1H-Cyclopropa[a]naphthalene, 1a,2,3,5,6,7,7a,7b-octahydro-1,1,7,7a-tetramethyl-, [1aR-(1a.alpha.,7.alpha.,7a.alpha.,7b.alpha.)]-	• cis-Calamenene	• 1-Undecene, 7-methyl-	• Bis(2-isopropyl-5-methylcyclohexyl) methylphosphonate (isomer 2)
	• (1S,4S,4aS)-1-Isopropyl-4,7-dimethyl-1,2,3,4,4a,5-hexahydronaphthalene	• Naphthalene, 1,2,3,4,4a,7-hexahydro-1,6-dimethyl-4-(1-methylethyl)-	• 4a(2H)-Naphthalenol, 1,3,4,5,6,8a-hexahydro-4,7-dimethyl-1-(1-methylethyl)-, (1S,4S,4aS,8aR)-	• 3,6-Dimethylpiperazine-2,5-dione
		• Caryophyllenyl alcohol		

Table S3. Cont.

SFM-CS131	SFM-confrontation	R5A-CS131	R5A-confrontation	Ew
	• Naphthalene, 1,2,3,5,6,8a-hexahydro-4,7-dimethyl-1-(1-methylethyl)-, (1S-cis)<br>• 1-Isopropyl-4,7-dimethyl-1,2,3,4,5,6-hexahydronaphthalene<br>• Bicyclosesquiphellandren<br>• cis-Muurola-4(15),5-diene<br>• cis-Calamenene<br>• Caryophyllenyl alcohol<br>• cis-Linaloloxide<br>• 4a(2H)-Naphthalenol, 1,3,4,5,6,8a-hexahydro-4,7-dimethyl-1-(1-methylethyl)-, (1S,4S,4aS,8aR)-	• 4a(2H)-Naphthalenol, 1,3,4,5,6,8a-hexahydro-4,7-dimethyl-1-(1-methylethyl)-, (1S,4S,4aS,8aR)-	• (1S,4S,4aS)-1-Isopropyl-4,7-dimethyl-1,2,3,4,4a,5-hexahydronaphthalene<br>• Naphthalene, 1,2,3,5,6,8a-hexahydro-4,7-dimethyl-1-(1-methylethyl)-, (1S-cis)-<br>• 1-Isopropyl-4,7-dimethyl-1,2,3,4,5,6-hexahydronaphthalene<br>• Bicyclosesquiphellandrene	

Table S4. Summary of compounds identified by GS-MS in the assay of Volatile Organic Compounds produced by the confrontation of *Streptomyces* CS131 and *E. weberi*. In the first column, VOCs exclusively detected on SFM confrontation; in the second, VOCs in common between SFM confrontation and SFM131

VOCs exclusively in CS131 SFM-confrontation	VOCs in common in SFM-confrontation and SFM-CS131
• 1,2,4,5-Tetrazin-3-amine<br>• cis-Linalool oxide<br>• Disulfide, bis(1,1,3,3-tetramethylbutyl)<br>• 2-Furanmethanol, 5-ethenyltetrahydro-alpha, alpha,5-trimethyl-, cis-<br>• 2-Methylenebornane<br>• 3-Octanone<br>• Bicyclo[3.1.0]hex-2-ene, 2-methyl-5-(1-methylethyl)-	• trans-Linalool oxide (furanoid)<br>• Tetrasulfide, dimethyl

Table S5. Compounds identified by GS-MS in the assay of Volatile Organic Compounds produced by the confrontation *Streptomyces* CS147 and *E. weberi* (Ew: volatiles produced by *E. weberi*; SFM-confrontation: volatiles produced during the confrontation *E.weberi* - *Streptomyces* CS147 on SFM; SFM-CS147: volatiles produced by CS147 on SFM; R5A-CS147: volatiles produced by CS147 on R5A; R5A-confrontation: volatiles produced during the confrontation *E.weberi* - *Streptomyces* CS147.

**SFM-CS147**	**SFM-confrontation**	**R5A-CS147**	**R5A-confrontation**	**Ew**
• Tricyclo[2.2.1.0(2,6)]heptane, 1,3,3-trimethyl-	• Tricyclo[2.2.1.0(2,6)]heptane, 1,3,3-trimethyl-	• Benzene, 1-ethoxy-4-ethyl-	• Bicyclo[3.1.0]hex-2-ene, 4-methyl-1-(1-methylethyl)-	• 2,6-Di-tert-butyl-4-hydroxy-4-methylcyclohexa-2,5-dien-1-one
• Camphene	• Camphene	• Bicyclo[3.1.0]hex-2-ene, 4 -methyl-1-(1-methylethyl)-	• Octane, 2-methyl-	• Isophthalicacid, 3,5-difluorophenyl heptylester
• 1,3-Cyclopentadiene, 1,3-bis(1-methylethyl)-	• 1,3-Cyclopentadiene, 1,3-bis(1-methylethyl)-	• p-Menth-8-ene, 3-methylene-	• Bicyclo[3.1.0]hexane, 4-methylene-1-(1-methylethyl)-	• Phosphoramidousdifluoride, dimethyl
• Dimethyl trisulfide	• Dimethyltrisulfide	• Decane, 4-methyl-	• Benzene, 1-ethoxy-4-ethyl-	• 3-Butene-1,2-diol
• Bicyclo[3.1.0]hex-2-ene, 4-methyl-1-(1-methylethyl)-	• 3-Octanone	• 4,8-dimethylnona-1,3,7-triene	• p-Menth-8-ene, 3-methylene-	• 1-Dodecanol
• Bicyclo[3.1.1]heptane, 6,6-dime thyl-2-methylene-, (1S)-	• Bicyclo[3.1.0]hex-2-ene, 4-methyl-1-(1-methylethyl)-	• 1,7,7-Trimethylbicyclo[2.2.1]hept-5-en-2-one	• 4,8-dimethylnona-1,3,7-triene	• Bicyclo[3.2.0]heptan-2-one, 6-hydroxy-5-methyl-6-vinyl
• 2-Methylenebornane	• Bicyclo[3.1.1]heptane, 6,6-dimethyl-2-methylene-, (1S)-	• 3-Oxabicyclo[5.3.0]decan-2-one, 9-methylene-, cis-	• 3-Oxabicyclo[5.3.0]decan-2-one, 9-methylene-, cis-	• Succinimide
• p-Menth-8-ene, 3-methylene-	• p-menth-8-ene, 3-methylene-	• 2-Methylisoborneol		

Table S5. Cont.

SFM-CS147	SFM-confrontation	R5A-CS147	R5A-confrontation	Ew
• Decane, 4-methyl-<br>• 4,8-dimethylnona-1,3,7-triene<br>• Octane, 5-ethyl-2-methyl-<br>• Ethanone, 1-[2-methyl-5-(1-meth ylethenyl)cyclopentyl]-, (1.alpha.,2.alpha.,5.beta.)-<br>• 3-Oxabicyclo[5.3.0]decan-2-one, 9-meth ylene-, cis-<br>• 3-Oxabicyclo[5.3.0]decan-2-one, 9-methylene-, trans-<br>• 2-Methylisoborneol<br>• 1H-Benzocycloheptene, 4,4a,5,6,7, 8,9,9a-octahydro-4a-methyl-, trans-	• 4,8-dimethylnona-1,3,7-triene<br>• 2-Methylenebornane<br>• 3-Oxabicyclo[5.3.0]decan-2-one, 9-methylene-, cis-<br>• 3-Oxabicyclo[5.3.0]decan-2-one, 9-methylene-, trans-5-(1-methylvinyl)-8-methyl-<br>• Liguloxide<br>• 1-Isopropyl-4,7-dimethyl-1,2,3,4,5,6-hexahydronaphthalene<br>• cis-Muurola-4(15),5-diene<br>• Bicyclosesquiphellandrene	• 1H-Indene, 1-ethylideneoctahydro-7a-methyl-, (1Z,3a.alpha.,7a.beta.)-<br>• 1-Cyclohexene-1-acetalde hyde, 2,6,6-trimethyl-<br>• Tricyclo[3.3.3.0]undecan-3-one<br>• 2-Caren-4-ol<br>• 2,4,4,6,6,8,8-Heptamethyl2 -2-nonene<br>• trans-1,10-Dimethyl-trans-9-decalinol<br>• 1H-Cyclopropa[a]naphthalene, 1a,2,3,5,6,7,7a,7b-octahydro-1,1,7,7a-tetramethyl-, [1aR-(1a.alpha.,7.alpha.,7a.alpha.,7b. alpha.)]-	• 2-Methylisoborneol<br>• 1-Cyclohexene-1-acetaldehyde, 2,6,6-trimethyl-<br>• Cyclohexanone, 2,5-dimethyl-2-(1-methylethenyl)-<br>• Tricyclo[3.3.3.0]undecan-3-one<br>• Decane, 2,3,6-trimethyl-<br>• Silane, diethylhexyloxytridecyloxy<br>• cis-Chrysanthenol	• Pentanoicacid, 5-hydroxy-, 2,4-di-t-butylphenyl esters<br>• 2-Methylisoborneol<br>• Heptadecane<br>• 2,4,4,6,6,8,8-Heptamethyl-2-nonene<br>• Pyrrolidine<br>• Pyrazine, 2,5-dimethyl<br>• Acetophenone

Table S5. Cont.

SFM-CS147	SFM-confrontation	R5A-CS147	R5A-confrontation	Ew
• 1H-Indene, 1-ethylideneoctahydro-7a-methyl-, (1Z,3a.alpha.,7a.beta.)-<br>• Cyclohexane, 1,1,4,4-tetramethyl-2, 5-dimethylene-<br>• 1-Cyclohexene-1-acetaldehyde, 2,6,6-trimethyl-<br>• Propanoic acid, 2,2-dimethyl-, anhydride with diethylborinic acid<br>• 2(1H)-Azulenone, 4,5,6,7,8,8a-hexahydro-8a-methyl-, (S)-<br>• Nonane, 5-(2-methylpropyl)-<br>• trans-1,10-Dimethyl-trans-9-decalinol	• 2H-3,9a-Methano-1-benzoxepin, octahydro-2,2,5a,9-tetramethyl-, [3R-(3.alpha.,5a.alpha.,9.alpha.,9a.alpha.)]-<br>• Naphthalene, 1,2,3,5,6,8a-hexahydro-4,7-dimethyl-1-(1-methylethyl)-, (1S-cis)-<br>• cis-Calamenene<br>• 2H-Benzimidazol-2-one, 1,3-dihydro-5-methoxy-<br>• Nonadecane, 2-methyl-<br>• 2-Methylisoborneol<br>• 1H-Indene, 1-ethylideneoctahydro-7a-methyl-, (1Z,3a.alpha.,7a.beta.)-	• (1S,4S,4aS)-1-Isopropyl-4,7-dimethyl-1,2,3,4,4a,5-hexahydronaphthalene<br>• Naphthalene, 1,2,3,5,6,7, 8,8a-octahydro-1,8a-dimethyl-7-(1-methylethenyl)-, [1R-(1 alpha.,7.beta.,8a.alpha.)]-<br>• Liguloxide<br>• 1R,2S,6S,7S,8S)-8-Isopropyl(-1-methyl-3-methylenetricyclo[4.4.0.02,7]decane-rel-<br>• 2-Bromotetradecane<br>• Bicyclosesquiphellandrene<br>• 1-Isopropyl-4,7-dimethyl1,2,3,4,5,6-hexahydronaphthalene-	• trans-1,10-Dimethyl-trans-9-decalinol<br>• 1H-Cyclopropa[a]naphthalene, 1a, 2,3,5,6,7,7a,7b-octahydro-1,1,7,7a-tetramethyl-, [1aR-(1a.alpha.,7.alpha.,7a.alpha.,7b.alpha.)]-<br>• Liguloxide<br>• Bicyclosesquiphellandrene<br>• Caryophyllenyl alcohol<br>• cis-Calamenene<br>• Decane, 3-ethyl-3-methyl-	• 2-Pentene, 2,4,4-trimethyl<br>• 3-Heptene, 2,2,4,6,6-pentamethyl<br>• Cyclohexane, 1-ethyl-1-methyl<br>• Hexane, 3,3-dimethyl<br>• Bis(2-isopropyl-5-methylcyclohexyl) methylphosphonate (isomer 2)<br>• 3,6-Dimethylpiperazine-2,5-dione

Table S5. Cont.

SFM-CS147	SFM-confrontation	R5A-CS147	R5A-confrontation	Ew
• 1H-Cyclopropa[a]naphthalene, 1a,2,3,5,6,7,7a,7b-octahydro-1,1,7,7a-tetramethyl-, [1aR-(1a.alpha.,7.alpha.,7a.alpha.,7b.alpha.)]-<br>• Azulene, 1,2,3,3a,4,5,6,7-octahydro-1,4-dimethyl-7-(1-methylethenyl)-, [1R-(1.alpha.,3a.beta.,4.alpha.,7.beta.)]-<br>• (1S,4S,4aS)-1-Isopropyl-4,7-dimethyl-1,2,3,4,4a,5-hexahydronaphthalene<br>• Bicyclo[5.3.0]decane, 2-methylene-5-(1-methylvinyl)-8-methyl-<br>• Liguloxide<br>• 2-Bromotetradecane<br>• Bicyclosesquiphellandrene	• Cyclohexane, 1,1,4,4-tetramethyl-2,5-dimethylene-<br>• 1-Cyclohexene-1-acetaldehyde, 2,6,6-trimethyl-<br>• 2(1H)-Azulenone, 4,5,6,7,8, 8a-hexahydro-8a-methyl-, (S)-<br>• trans-1,10-Dimethyl-trans-9-decalinol<br>• 1H-Cyclopropa[a]naphthalene, 1a,2,3,5,6,7,7a,7b-octahydro-1,1,7,7a-tetramethyl-, [1aR-(1a.alpha.,7.alpha.,7a.alpha.,7b.alpha.)]-<br>• (1S,4S,4aS)-1-Isopropyl-4,7-dimethyl-1,2,3,4,4a,5-hexahydronaphthalene<br>• Bicyclo[5.3.0]decane, 2-methylene-2,7-Cyclodecadiene-1-methanol, .alpha.,.alpha.,4,8-tetramethyl-	• Naphthalene, 1,2,3,5,6,8a-hexahydro-4,7-dimethyl-1-(1-methylethyl)-, (1S-cis)-<br>• cis-Calamenene<br>• Caryophyllenyl alcohol<br>• 4a(2H)-Naphthalenol, 1,3,4, 5,6,8a-hexahydro-4,7-dimet hyl-1-(1-methylethyl)-, (1S,4S,4aS,8aR)-<br>• 2,7-Cyclodecadiene-1-methanol, .alpha.,.alpha.,4,8-tetramethyl-<br>• 1-Benzyloxy-1-ethyl-1-silacyclopentane	• 4a(2H)-Naphthalenol, 1,3,4,5,6,8a-hexahydro-4,7-dimethyl-1-(1-methylethyl)-, (1S,4S,4aS,8aR)-<br>• 2,7-Cyclodecadiene-1-methanol, alpha.,.alpha.,4,8-tetramethyl-<br>• Isophthalic acid, ethyl tridec-2-ynyl ester<br>• 1-Benzyloxy-1-ethyl-1-silacyclopentane<br>• Isophthalic acid, di(2-fluorophenyl) ester	

Table S5. Cont.

SFM-CS147	SFM-confrontation	R5A-CS147	R5A-confrontation	Ew
• 2H-3,9a-Methano-1-benzoxepin, octahydro-2,2,5a,9-tetramethyl-, [3R-(3.alpha.,5a.alpha.,9.alpha.,9a.alpha.)]-<br>• cis-Calamenene<br>• Naphthalene, 1,2,3,5,6,8a-hexahydro<br>• -4,7-dimethyl-1-(1-methylethyl)-, (1S-cis)-<br>• 4a(2H)-Naphthalenol, 1,3,4,5,6,8a-hexahydro-4,7-dimethyl-1-(1-methylethyl)-, (1S,4S,4aS,8aR)-<br>• 4H-Benzo[b]pyrane-3-carbonitrile, 5,6,7,8-tetrahydro-2-amino-4-(3-cyclohexenyl)-7,7-dimethyl-5-oxo<br>• Isophthalic acid, di(2-fluorophenyl) ester	• 4a(2H)-Naphthalenol, 1,3,4,5,6,8a-hexahydro-4,7-dimethyl-1-(1-methylethyl)-, (1S,4S,4aS,8aR)-<br>• 3,5-Di-tert-butyl-2-hydroxybenzaldehyde	• 2-Amino-4-(2-cyclohexyl-ethyl)-7<br>• -methyl-5-oxo-4H,5H-pyrano[4,3-b]pyran-3-carbonitrile		

Table S6. Summary of compounds identified by GS-MS in the assay of Volatile Organic Compounds produced by the confrontation of *Streptomyces* CS147 and *E. weberi*. In the first column, VOCs exclusively detected on SFM confrontation, in the second; VOCs in common between SFM confrontation and SFM147

VOCs exclusively in CS147 SFM-confrontation	VOCs in common in SFM-confrontation and SFM-CS147
• 3-Octanone<br>• cis-Muurola-4(15),5-diene<br>• 3,5-Di-tert-butyl-2-hydroxybenzaldehyde<br>• Nonadecane, 2-methyl-<br>• 2H-Benzimidazol-2-one, 1,3-dihydro-5-methoxy-	• Tricyclo[2.2.1.0(2,6)]heptane, 1,3,3-trimethyl-<br>• Camphene<br>• 1,3-Cyclopentadiene, 1,3-bis(1-methylethyl)-<br>• Dimethyl trisulfide<br>• Bicyclo[3.1.1]heptane, 6,6-dimethyl-2-methylene-, (1S)-<br>• 2-Methylenebornane<br>• 3-Oxabicyclo[5.3.0]decan-2-one, 9-methylene-, trans-<br>• Cyclohexane, 1,1,4,4-tetramethyl-2,5-dimethylene-<br>• 2(1H)-Azulenone, 4,5,6,7,8,8a-hexahydro-8a-methyl-, (S)-<br>• Bicyclo[5.3.0]decane, 2-methylene-5-(1-methylvinyl)-8-methyl-<br>• 2H-3,9a-Methano-1-benzoxepin, octahydro-2,2,5a,9-tetramethyl-, [3R-(3.alpha.,5a.alpha.,9.alpha.,9a.alpha.)]-

Table S7. Compounds identified by GS-MS in the assay of Volatile Organic Compounds produced by the confrontation *Streptomyces* CS014 and *E. weberi* (Ew: volatiles produced by *E. weberi*; SFM-confrontation: volatiles produced during the confrontation *E.weberi - Streptomyces* CS014 on SFM; SFM-CS014: volatiles produced by CS14 on SFM; R5A-CS014: volatiles produced by CS014 on R5A; R5A-confrontation: volatiles produced during the confrontation *E.weberi - Streptomyces* CS014.

SFM-CS014	SFM-confrontation	R5A-CS014	R5A-confrontation	Ew
• 2(3H)-Furanone, dihydro-3-methyl-<br>• Dimethyl trisulfide<br>• Heptane, 4-ethyl-<br>• Decane, 2,4-dimethyl-<br>• Tetrasulfide, dimethyl<br>• 1H-Indene, 1-ethylideneoctahydro-7a-methyl-, (1Z,3a.alpha.,7a.beta.)-<br>• Cyclohexane, 1,1,4,4-tetramethyl-2,5-dimethylene-	• alpha-Calacorene<br>• Cubenene<br>• cis-Calamenene<br>• Naphthalene, 1,2,3,5,6,8a-hexahydro-4,7-dimethyl-1-(1-methylethyl)-, (1S-cis)-<br>• 2H-3,9a-Methano-1-benzoxepin, octahydro-2,2,5a,9-tetramethyl-, [3R-(3.alpha.,5a.alpha.,9.alpha.,9a.alpha.)]-<br>• .alpha.-Muurolene<br>• cis-Muurola-4(15),5-diene	• Dimethyl trisulfide<br>• Bicyclo[4.3.0]non-3-ene, 3,4,7-trimethyl-<br>• 1H-Indene, 1-ethylideneoctahydro-7a-methyl-, (1Z,3a.alpha.,7a.beta.)-<br>• Cyclohexane, 1,1,4,4-tetramethyl-2,5-dimethylene-<br>• Cyclohexane, 1,1,4,4-tetramethyl-2,6-bis(methylene)-<br>• 2,4,4,6,6,8,8-Heptamethyl-2-nonene	• 1H-Indene, 1-ethylideneoctahydro-7a-methyl-, (1Z,3a.alpha.,7a.beta.)-<br>• Decane, 2,3,5-trimethyl-<br>• Decane, 1-iodo-<br>• 1-Tetradecene<br>• 1H-Cyclopropa[a]naphthalene, 1a,2,3,5,6,7,7a,7b-octahydro-1,1,7,7a-tetramethyl-, [1aR-(1a.alpha.,7.alpha.,7a.alpha.,7b.alpha.)]-<br>• trans-1,10-Dimethyl-trans-9-decalinol	• 2,6-Di-tert-butyl-4-hydroxy-4-methylcyclohexa-2,5-dien-1-one<br>• Isophthalicacid, 3,5-difluorophenyl heptylester<br>• Phosphoramidousdifluoride, dimethyl<br>• 3-Butene-1,2-diol<br>• 1-Dodecanol<br>• Bicyclo[3.2.0]heptan-2-one, 6-hydroxy-5-methyl-6-vinyl<br>• Succinimide

Table S7. Cont.

SFM-CS014	SFM-confrontation	R5A-CS014	R5A-confrontation	Ew
• Bicyclo[4.3.0]non-3-ene, 3,4,7-trimethyl<br>• Octane, 1-iodo-<br>• 2(1H)-Azulenone, 4,5,6,7,8,8a-hexahydro-8a-methyl-, (S)-<br>• Cyclopropanebutanoic acid, 2,4-dioxo-, methyl ester<br>• Cubenene<br>• trans-1,10-Dimethyl-trans-9-decalinol<br>• Azulene, 1,2,3,3a,4,5,6,7-octahydro-1,4-dimethyl-7-(1-methylethenyl)-, [1R-(1.alpha.,3a.beta.,4.alpha.,7.beta.)]<br>• 1H-Cyclopropa[a]naphthalene, 1a,2,3,5,6,7,7a,7b-octahydro-1, 1,7,7a-tetramethyl-, [1aR-(1a.alpha.,7.alpha.,7a.alpha.,7b.alpha.)]-	• 1-Isopropyl-4,7-dimethyl-1,2,3,4,5,6-hexahydronaphthalene<br>• Liguloxide<br>• (1S,4S,4aS)-1-Isopropyl-4,7-dimethyl-1,2,3,4,4a,5-hexahydronaphthalene<br>• 1H-Cyclopropa[a]naphthalene, 1a,2,3,5,6,7,7a,7b-octahydro-1,1,7,7a- tetramethyl-, [1aR-(1a.alpha.,7.alpha.,7a.alpha.,7b.alpha.)]-<br>• Azulene, 1,2,3,3a,4,5,6,7-octahydro-1,4-dimethyl-7-(1-methylethenyl)-, [1R-(1.alpha.,3a.beta.,4.alpha.,7.beta.)]-<br>• trans-1,10-Dimethyl-trans-9-decalinol<br>• Dodecane, 5-methyl-	• trans-1,10-Dimethyl-trans-9-decalinol<br>• (-)-Aristolene<br>• 1H-Cyclopropa[a]naphthalene, 1a,2,3,5,6,7,7a,7b-octahydro-1,1,7,7a-tetramethyl-, [1aR-(1a.alpha.,7.alpha.,7a.alpha.,7b.alpha.)]-<br>• 1,4,7,-Cycloundecatriene, 1,5,9,9-tetramethyl-, Z,Z,Z-<br>• 1-Isopropyl-4,7-dimethyl-1,2,3,4,5,6-hexahydronaphthalene<br>• cis-Muurola-4(15),5-diene<br>• .alpha.-Muurolene	• 5-Methyl-2,4-diisopropylphenol<br>• 1-Isopropyl-4,7-dimethyl-1,2,3,4,5,6-hexahydronaphthalene<br>• .alpha.-Muurolene<br>• cis-Calamenene<br>• Naphthalene, 1,2,3,5,6,8a-hexahydro-4,7-dimethyl-1-(1-methylethyl)-, (1S-cis)-	• Pentanoicacid, 5-hydroxy-, 2,4-di-t-butylphenyl esters<br>• 2-Methylisoborneol<br>• Heptadecane<br>• 2,4,4,6,6,8,8-Heptamethyl-2-nonene<br>• Pyrrolidine<br>• Pyrazine, 2,5-dimethyl<br>• Acetophenone

Table S7. Cont.

SFM-CS014	SFM-confrontation	R5A-CS014	R5A-confrontation	Ew
• (1S,4S,4aS)-1-Isopropyl-4,7-dimethyl-1,2,3,4,4a,5-hexahydronaphthalene<br>• Naphthalene, 1,2,3,5,6,8a-hexahydro-4,7-dimethyl-1-(1-methylethyl)-, (1S-cis)-<br>• Liguloxide<br>• cis-Muurola-4(15),5-diene<br>• Methyl (2R,3R,4S)-3-(tert-butyldimethylsilyloxy)-2,4-dimethylhexanoate<br>• Octahydroxanthen-1,9-dione, 3, 3,7,7,-tetramethyl-10-hexyl-	• 2(1H)-Azulenone, 4,5,6,7,8,8a-hexahydro-8a-methyl-, (S)-<br>• Cyclohexane, 1,1,4,4-tetramethyl-2,5-dimethylene-<br>• 1H-Indene, 1-ethylideneoctahydro-7a-methyl-, (1Z,3a.alpha.,7a.beta.)-<br>• Tetrasulfide, dimethyl<br>• Heptane, 4-ethyl-<br>• 3-Octanone<br>• Dimethyl trisulfide<br>• 2(3H)-Furanone, dihydro-3-methyl	• Naphthalene, 1,2,3,5,6,8a-hexahydro-4,7-dimethyl-1-(1-methylethyl)-, (1S-cis)-<br>• cis-Calamenene<br>• Cubenene<br>• Methyl (2R,3R,4S)-3-(tert-butyldimethylsilyloxy)-2,4-dimethylhexanoate		• 2-Pentene, 2,4,4-trimethyl<br>• 3-Heptene, 2,2,4,6,6-pentamethyl<br>• Cyclohexane, 1-ethyl-1-methyl<br>• Hexane, 3,3-dimethyl<br>• Bis(2-isopropyl-5-methylcyclohexyl) methylphosphonate (isomer 2)<br>• 3,6-Dimethylpiperazine-2,5-dione

Table S8. Summary of compounds identified by GS-MS in the assay of Volatile Organic Compounds produced by the confrontation of *Streptomyces* CS014 and *E. weberi.* In the first column, VOCs exclusively detected on SFM confrontation; in the second, VOCs in common between SFM confrontation and SFM14

VOCs exclusively in CS014 SFM-confrontation	VOCs in common in SFM-confrontation and SFM-CS014
• alpha-Calacorene<br>• 2H-3,9a-Methano-1-benzoxepin, octahydro-2,2,5a,9-tetramethyl-, [3R-(3.alpha.,5a.alpha.,9.alpha.,9a.alpha.)]-<br>• Dodecane, 5-methyl-<br>• 3-Octanone	• Liguloxide<br>• (1S,4S,4aS)-1-Isopropyl-4,7-dimethyl-1,2,3,4,4a,5-hexahydronaphthalene<br>• Azulene, 1,2,3,3a,4,5,6,7-octahydro-1,4-dimethyl-7-(1-methylethenyl)-, [1R-(1.alpha.,3a.beta.,4.alpha.,7.beta.)]-<br>• 2(1H)-Azulenone, 4,5,6,7,8,8a-hexahydro-8a-methyl-, (S)-<br>• Tetrasulfide, dimethyl<br>• Heptane, 4-ethyl-<br>• 2(3H)-Furanone, dihydro-3-methyl-
